# Chlorine (Cl) and hydrogen chloride (HCl) solubility in hydrous silicate melts: implications for volcanic gas composition

**DOI:** 10.1007/s00410-026-02332-x

**Published:** 2026-05-22

**Authors:** Monika K. Rusiecka, Bernard J. Wood

**Affiliations:** Department of Earth Sciences, University of Oxford, South Parks Road, Oxford, OX1 3AN UK

## Abstract

Chlorine is a key volatile in magmatic and volcanic systems, being a major consumer of atmospheric ozone and an important ligand for metal transport. Despite chlorine’s importance however, the combined effects of melt composition and H₂O on Cl solubility and HCl degassing remain poorly constrained, particularly at magmatic pressures. We present high-pressure (0.5–1.63 GPa), 1200 °C experiments on hydrous andesite, dacite and rhyolite (up to ~ 8–9 wt% H₂O) equilibrated with Ag–AgI–AgCl buffers to fix fCl₂, and with graphite-saturated C–O–H fluids to buffer fO₂ near CCO. Chlorine concentrations in the glasses define chloride capacities (C_Cl_) that we combine with anhydrous data from earlier work. Using LASSO regression, we obtain a revised chloride-capacity model that improves predictions for evolved, silica- and alkali-rich melts. The explicit HO_0.5_ term remains statistically insignificant up to ~ 8–9 wt% H₂O, confirming that H₂O acts primarily as an ideal diluent of network-forming and network-modifying cations rather than exerting a strong, specific control on Cl dissolution. We couple the new C_Cl_ model with H₂–H₂O–HCl equilibria and a C–H–O–S–Cl fluid speciation code to derive a simple expression for HCl fugacity in terms of melt composition, temperature, pressure and fH₂O, without requiring explicit values of fCl₂ or fO₂. Applied to literature experiments and melt inclusions, the model predicts (i) low fCl₂ (~ 10^− 10^–10^− 6^ bar) and much higher fHCl (~ 10⁻^1^–10^2^ bar) at near-surface pressures, consistent with HCl-dominated volcanic emissions; (ii) SO₂/HCl ratios for the 2002–2003 Mt. Etna eruption that match plume measurements; and (iii) strong variations in SO₂/HCl and S/Cl during decompression due to contrasting pressure dependences of sulphur and chlorine capacities. Our framework links petrological archives (melt inclusions, matrix glasses) to HCl contents of magmatic gases, providing a tool to interpret excess degassing, magma plumbing and volcanic halogen fluxes to the atmosphere. We also used HSC Chemistry software to calculate the production of reactive chlorine species (ClO) from HCl in volcanic gases upon mixing with air at the vent.

**Supplementary Information:**

The online version contains supplementary material available at 10.1007/s00410-026-02332-x.

## Introduction

Volatile components drive the evolution of magmatic systems. Of these volatiles, H₂O and CO₂ dominate because they are the most abundant, they strongly affect melting and crystallisation, and they control volcanic degassing and ore formation (Symonds et al. 1994; De Vivo et al. 2005). CO₂ gained attention when improved gas-sampling methods revealed its role in the atmospheric greenhouse budget (Heald et al. 1963; Gerlach and Nordlie 1975). As a result, extensive solubility datasets and models now exist for both H₂O and CO₂ (Moore et al. 1998; Newman and Lowenstern 2002; Ghiorso and Gualda 2015; Brooker et al. 2001; Stanley et al. 2011).

Volatile species of sulphur, have been the focus of considerable attention recently because sulphur is also abundant in volcanic gases, sensitive to redox conditions, and soluble as both S²⁻ and S⁶⁺ depending on oxygen fugacity (Fincham and Richardson 1954). Recent experiments have constrained its speciation and solubility under diverse conditions, enabling accurate predictions of degassing and redox evolution (O’Neill and Mavrogenes, 2002; Boulliung and Wood 2022, 2023; Ding et al. 2023). Halogens, although less abundant, are geologically and environmentally important. Volcanic gases contain HCl and HF in the 0.1–10% range, with trace HBr and HI (Pyle and Mather 2009; Martin et al. 2012). Current global emissions reach up to 5.5 Tg/yr of HCl and 0.15–0.58 Tg/yr of HF (Gerlach 2004; Aiuppa et al. 2009; Webster et al. 2018). These emissions affect the atmosphere and biosphere. Cl-bearing gases transport metals such as Cu, Cd, and Pb (Scholtysik and Canil 2021), destroy stratospheric ozone (von Glasow et al. 2009), and cause severe local impacts, as during the 1783 Laki eruption in Iceland. Beyond environmental effects, halogens act as ligands in hydrothermal solutions, mobilising metals such as Cu and Au in ore-forming systems (e.g., Candela and Holland 1986; Blundy et al. 2015).

Experimental work shows that halogens alter both the physical and chemical properties of silicate melts. Cl lowers liquidus temperatures more efficiently than H₂O on a molar basis (Filiberto and Treiman 2009), and its solubility in melts varies strongly with composition, especially with CaO and other network-modifying cations (Signorelli and Carroll 2000; Webster and De Vivo 2002; Thomas and Wood 2023). Recent models quantify these effects (Webster et al. 2015), but major uncertainties remain because chlorine fugacity is generally poorly constrained in magmatic systems.

To address the quantification of chlorine behaviour in melts, Thomas and Wood (2021) developed a method for buffering chlorine fugacity using the Ag–AgCl reaction. In order to generate a range of chlorine fugacities at any given pressure and temperature they diluted the AgCl with varying amounts of AgI, generating AgI-AgCl melts in equilibrium with liquid Ag and silicate melt. This technique enables wide compositional control while limiting Ag dissolution into the silicate melt. Their experiments showed that Cl solubility follows Henry’s Law to high concentrations (> 2.5% Cl in basalt) with appropriate dependence on fCl₂ and fO₂, and that “chloride capacity” can be parameterised in terms of melt composition, pressure, and temperature (Thomas and Wood 2021, 2023). Spectroscopic studies confirm that Ca, Mg, Fe, and Na shape Cl bonding in silicate melts (Sandland et al. 2004; Stebbins and Du 2002; Thomas et al. 2023).

The experiments of Thomas and Wood (2021, 2023) addressed the fugacity of chlorine in anhydrous silicate melts. Volcanic degassing, however generally involves loss of halogens in an H_2_O-rich gas, which raises the question of the influence of H_2_O on chlorine solubility and behaviour. In order to determine the influence of dissolved H_2_O on Cl_2_ fugacity Rusiecka and Wood (2025) added up to 4 wt% H₂O to a natural basalt, which they equilibrated with the AgI–AgCl-Ag buffer at oxygen fugacities controlled by C–CO–CO₂ mixtures and pressures and temperatures of 0.5–1.63 GPa and 1200–1300 °C respectively. Their results showed that Cl contents increase with H₂O content, but only because H₂O dilutes the negative influence of network-forming cations such as SiO₂. Thus, H₂O appears to behave as a “neutral diluent,” with no direct effect on Cl dissolution. When combined with earlier anhydrous datasets, these experiments refined the chloride capacity model and confirmed that, even though H₂O exerts measurable effects on Cl behaviour, its effect is purely due to “ideal” dilution at up to 4 wt% in basalt.

H₂O is clearly the dominant volatile in magmas and volcanic gases, but its effects on Cl solubility, speciation and volatility are still poorly constrained for most melts of geologic importance. As discussed above, Rusiecka and Wood (2025) showed that in basaltic melts with up to 4 wt% H₂O, H_2_O behaves as an “ideal” diluent but whether this behaviour persists in more evolved magmas or at higher H₂O contents is unknown. In order to expand our understanding of the effects of H_2_O on Cl_2_ fugacity we have extended the approach of Rusiecka and Wood to andesite, dacite, and rhyolite compositions and to higher water concentrations (> 4 weight%). Our goals were twofold: (1) to determine whether H₂O remains an ideal diluent across a broad compositional spectrum, and (2) to assess how increasing H₂O content affects Cl incorporation in melts and partitioning into exsolved fluids during degassing.

## Experimental procedure

We conducted a series of experiments using synthetic starting materials with the composition of andesite (Carmichael et al. 1974), dacite from Brothers Volcano (Kermadec arc, de Ronde et al. 2019), and rhyolite from the Oruanui eruption of Taupō volcano (NZ, Sharpe et al. 2022).

Table 1 shows the anhydrous compositions of the starting materials. To make the starting material, we mixed analytical grade oxides (SiO₂, TiO₂, MgO, Al₂O₃) with carbonates (Na₂CO₃, CaCO₃).

**Table 1:** Anhydrous compositions of the starting materials used in this study

	SiO_2_	TiO_2_	Al_2_O_3_	FeO_tot_	MgO	CaO	Na_2_O	K_2_O
AND	62.49	0.62	18.20	3.99	3.47	6.85	3.35	1.03
HSR	77.30	0.13	12.77	1.62	0.12	1.02	3.52	3.46
DCB	66.76	1.02	14.62	5.36	1.42	4.02	4.64	2.16
DCB-Al	64.49	1.01	22.65	1.24	1.67	4.14	3.33	1.47

*AND* andesite (Carmicheal et al., 1974), *DCB* dacite from Brothers volcano (Kermadec arc, de Ronde et al., 2019), *DCB-Al *DCB with high alumina content, *HSR* high silica rhyolite from the Oruanui eruption of Taupō volcano (NZ, Sharpe et al. 2022)

We ground the powders under ethanol for one hour, then decarbonated them overnight by heating from 400 to 800 °C at 2 °C/min. After decarbonation, we added water as Al(OH)₃ and iron as Fe_0.95_O. We then reground the powders under isopropanol for at least an hour.

We controlled chlorine fugacity by mixing AgI and AgCl in an 80:20 ratio following Thomas and Wood (2021). Chlorine fugacity was calculated as a function of pressure (P, GPa) and temperature (T, K):

1 $$ \begin{aligned} \:{\mathrm{logf}}Cl_{2} & = \: - \frac{{1140}}{T} + 2.961 + 2150\frac{P}{T} - 208\frac{{P^{2} }}{T} \\ & + 2{\mathrm{log}}\left[ {\frac{{Cl}}{{Cl + I}}} \right]_{{halide}} - 2log\left[ {\frac{{Ag}}{{Ag + Pt}}} \right]_{{metal}} \\ \end{aligned} $$

**Fig. 1:**
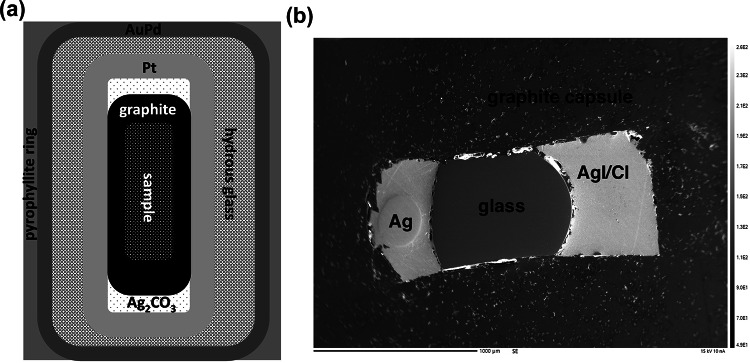
**a** Diagram of the triple-capsule experimental setup; **b** SE (secondary electron) image of the sample following the experiment showing glass, the AgI/Cl buffer material, and metallic silver

We mixed the starting silicate compositions with the AgI/AgCl buffer in a 75:25 buffer: silicate ratio, then packed them into graphite capsules (2.5 mm OD, 1 mm ID). We placed the graphite capsules, with graphite lids, above a layer of Ag₂CO₃ inside 3 mm OD Pt capsules. During the experiment, Ag₂CO₃ decomposes to metallic Ag, CO₂, and O₂, buffering oxygen fugacity close to the CCO buffer (depending on H₂O content) through reaction with the graphite container. We sealed the Pt capsule inside a 4 mm OD Au₈₀Pd₂₀ capsule, packed with the starting silicate material plus graphite. Figure 1a shows the triple-capsule experimental setup, whereas Fig. 1b displays a SE (secondary electron) image of the sample following the experiment. Following the procedure of Rusiecka and Wood (2025), we carried out the experiments in an end-loaded ½-inch piston-cylinder apparatus at the University of Oxford. At 1.05 and 1.63 GPa we used CaF₂ as the pressure medium; at 0.5 GPa we used a NaCl-borosilicate glass assembly. The 4 mm OD Au₈₀Pd₂₀ capsule was placed in the centre of a graphite furnace inside a 6 mm OD pyrophyllite sleeve, with crushable MgO spacers above and below.

Temperature was monitored and controlled throughout the experiment using a thermocouple. At 1.05 and 1.63 GPa, a C-type thermocouple sheathed in alumina was employed. At 0.5 GPa, we used a welded S-type thermocouple sheathed in mullite. In both cases, the thermocouple entered through a hole in the MgO spacer above the capsule and was separated from the capsule by a 0.5 mm thick alumina disc.

Thomas and Wood (2021) found that, in experiments performed at 1.6 GPa/1400°C for up to 2 h, the Cl content of the glass did not change significantly after 5 min at pressure and temperature. The Cl content is essentially established as the buffer separates from the silicate. Using these observations as a guide we used experiment durations of 1 (andesite) to 3 (rhyolite) hours. Analyses of product glasses indicates that their Cl contents are homogeneous within 2 standard deviations of the mean.

### Analytical procedure

The experimental run product capsules were mounted in epoxy resin and ground to expose the charges inside the inner graphite capsules. We polished the samples with baby oil as lubricant and oil-based diamond suspensions to prevent chlorine loss. The charges contained glass, Ag-rich metal blobs, and a metal chloride–iodide phase.

We analysed the silicate glasses, metal chloride–iodide, and metal blobs using the CAMECA SXFive-FE electron microprobe at the Department of Earth Sciences, University of Oxford. For glass and chloride–iodide, we used an accelerating voltage of 15 kV, a 4 nA beam current, and a 20 μm defocused beam. For metal blobs, we used 15 kV, 20 nA, and a 1 μm focused beam. Calibration employed albite (Si, Al, Na), wollastonite (Ca), fayalite (Fe in silicate glass), elemental Fe (Fe in metal and chloride–iodide), thallium bromoiodide (I), sanidine (K), periclase (Mg), TiO₂, elemental Ag and Pt, and high-purity synthetic NaCl (Cl). Secondary standards included St. John’s Island olivine and labradorite. Counting times were 30 s on peaks and 15 s on backgrounds, except for Cl (60 s peak, 30 s background), Mg (40 s peak, 20 s background), and Na (20 s peak, 10 s background).

The chloride-iodide phase was generally very soft and difficult to analyse but where accurate analyses were obtained, they indicated Na and K contents < 0.3%, Ca and Fe contents below detection and Mg ~ 0.5%. Assuming a Temkin solution of these cations in Ag(Cl, I) would change the activity of AgCl by < 2%, an insignificant amount (Rusiecka and Wood 2025) so they have been ignored in calculating Ag(Cl, I) composition by mass balance.

We measured H₂O, CO₂, Si, and Mg in samples mounted in gold-coated epoxy mounts using the Cameca IMS-7f GEO at the NERC Ion Microprobe Facility, University of Edinburgh. A ¹⁶O⁻ primary beam with 3 nA current and 18 kV impact energy produced a ~ 10 μm spot. We normalised raw counts of ¹H, ¹²C, and ²⁶Mg to ³⁰Si count rates. We then converted normalised counts to H₂O and CO₂ contents using calibration curves based on well-characterised basaltic and rhyolitic glass standards (N72, M47, M10, M5, M21, M36, M40, Shishkina et al. 2010; 519-4-1, Hauri 2002; RB480; Brooker et al. 1999; Run #48, #51, #59, #61, Mangan and Sisson 2000; ND70 glasses, Moussallam et al. 2024). As H_2_O showed a strong matrix effect between rhyolite and basalt, we calculated interpolated calibration curves based on the SiO_2_ content of the experiments. H_2_O and CO_2_ background signals were estimated based on San Carlos olivine mounted with the experimental glasses and subtracted from the signals, and amounted to ca. 0.015 wt% H_2_O and 35 ppmw CO_2_ only.

## Results

We performed fourteen experiments at 1200 °C and pressures of 0.5, 1.05, and 1.63 GPa on hydrous andesite, Brothers volcano dacite, and Oruanui high-silica rhyolite. These experimental conditions, combined with our experimental set-up, limited hydrogen diffusion and water loss from the capsules. All experiments used the same AgI/AgCl (80/20) ratio in the buffer and buffer/silicate (75/25) ratio in the capsule.

We buffered oxygen fugacity (fO₂) near C–CO–CO₂ (CCO) using graphite in equilibrium with a C–O-(H) fluid. At 1.05 and 1.63 GPa, we calculated fO₂ in anhydrous experiments using the Jakobsson and Oskarsson (1994) equation. At 0.5 GPa, where that model was not calibrated, we used the Modified Redlich–Kwong (MRK) equation of state (Holloway 1977), assuming, in anhydrous experiments that the fluid contained only CO and CO₂ in equilibrium with graphite.

To test the MRK approach, we calculated fO₂ under the conditions used by Jakobsson and Oskarsson (1994). We combined NIST–JANAF (Chase 1998) 1-bar free energies for CO and CO₂ with Holloway’s MRK formulation, keeping the standard state at 1 bar. We then applied MRK mixing rules for CO–CO₂ and calculated fO₂ from the equilibria:

- C(graphite) + O₂ = CO₂
- 2 C(graphite) + O₂ = 2CO

For these calculations, we used a graphite molar volume of 5.288 cm³ at 298 K (expanded to 1200 °C at 1 bar; Zhao et al. 2022) and integrated to experimental pressures with a constant compressibility of 0.034 GPa⁻¹ (Hanfland et al. 1989). We adjusted the CO/CO₂ ratio until the fO₂ calculated from C-CO_2_ equilibrium agreed with that obtained from C-CO equilibrium. At 1.05 and 1.63 GPa, MRK matched experimental results calculated using the equation of Jakobsson and Oskarsson (1994) within 0.05 log units, supporting the use of MRK at 0.5 GPa.

For hydrous experiments, we converted glass H₂O contents to fugacities using the solubility equations of Burnham et al. (1994). We then used MRK to translate H₂O fugacity into mole fractions and fugacity coefficients for CO, CO₂, H₂O, H₂, and CH₄ at the experimental fO₂. Finally, we calculated fO₂ at graphite saturation in the presence of H₂O using the CO and CO₂ fugacity coefficients (all fO₂ in the experiments are listed in Table 2).

**Table 2:** Experimental conditions and measured compositions in the experiments conducted in this study

ID	T (°C)	*P* (GPa)	Final Molar Cl/(Cl + I)	Molar Ag/(Ag + Pt)	fCl_2_	fO_2_	fHCl	fCl_2_/fHCl	C_Cl_
AND-1	1200	1.05	0.27	0.99	2.80E-05	5.54E-10	1563	1.79E-08	1.68
HSR-1	1200	1.63	0.29	0.99	1.34E-04	2.61E-09	435	3.09E-07	0.11
AND-2	1200	1.63	0.28	0.99	1.23E-04	2.61E-09	2268	5.41E-08	0.90
AND-3	1200	1.63	0.28	0.99	1.23E-04	2.61E-09	2126	5.81E-08	0.85
AND-4	1200	1.63	0.28	0.99	1.24E-04	2.61E-09	1184	1.05E-07	0.80
AND-5	1200	1.05	0.28	0.99	2.91E-05	5.54E-10	809	3.60E-08	1.18
AND-6	1200	1.05	0.27	0.99	2.87E-05	5.54E-10	1471	1.95E-08	1.38
DCB-1	1200	1.05	0.28	0.99	2.93E-05	5.54E-10	1655	1.77E-08	1.13
DCB-2	1200	1.63	0.28	0.99	1.23E-04	2.61E-09	2741	4.48E-08	0.89
DCB-Al-1	1200	1.05	0.28	0.99	3.04E-05	5.54E-10	744	4.09E-08	0.67
DCB-3	1200	1.05	0.28	0.99	3.01E-05	5.54E-10	999	3.02E-08	0.78
DCB-4	1200	1.63	0.28	0.99	1.26E-04	2.61E-09	2003	6.27E-08	0.70
HSR-2	1200	1.05	0.29	0.99	3.11E-05	5.54E-10	1058	2.94E-08	0.40
HSR-3	1200	1.63	0.28	0.99	1.31E-04	2.61E-09	1949	6.72E-08	0.34
AND-7	1200	0.5	0.28	0.99	6.08E-06	4.44E-11	476	1.28E-08	1.32
DCB-5	1200	0.5	0.28	0.99	6.32E-06	4.21E-11	381	1.66E-08	0.76

P – pressure in GPa; T – temperature in °C; fCl_2_ – chlorine fugacity; fO_2_ – oxygen fugacity; fHCl – hydrogen chloride fugacity; C_Cl_ – chloride capacity

**Table 3:** Measured compositions of the experimental samples

ID	*n*	SiO_2_	TiO_2_	Al_2_O_3_	FeO_tot_	MgO	CaO	Na_2_O	K_2_O	Cl	CO_2_	H_2_O	Total
AND-1	31	56.16 ± 0.65	0.56 ± 0.02	14.47 ± 0.35	2.77 ± 0.11	7.89 ± 0.54	6.16 ± 0.12	1.97 ± 0.10	0.48 ± 0.05	1.83 ± 0.04	0.41 ± 0.022	7.18 ± 0.13	100.3
HSR-1	5	73.27 ± 0.42	0.08 ± 0.02	11.75 ± 0.44	1.45 ± 0.05	0.28 ± 0.04	1.1 ± 0.08	3.11 ± 0.15	2.93 ± 0.08	0.18 ± 0.01	0.68 ± 0.227	4.81 ± 0.07	100.0
AND-2	23	58.44 ± 0.67	0.60 ± 0.02	14.67 ± 0.25	3.35 ± 0.11	3.42 ± 0.11	6.65 ± 0.13	2.52 ± 0.11	0.69 ± 0.05	1.39 ± 0.03	0.81 ± 0.040	6.87 ± 0.10	99.8
AND-3	19	57.45 ± 0.55	0.58 ± 0.03	15.49 ± 0.24	3.4 ± 0.12	3.16 ± 0.10	6.34 ± 0.18	2.43 ± 0.16	0.68 ± 0.06	1.32 ± 0.03	1.03 ± 0.030	7.23 ± 0.18	99.4
AND-4	20	60.32 ± 0.59	0.60 ± 0.02	15.79 ± 0.25	3.47 ± 0.10	3.26 ± 0.10	6.55 ± 0.14	2.59 ± 0.13	0.80 ± 0.04	1.24 ± 0.02	0.46 ± 0.093	4.62 ± 0.08	100.1
AND-5	14	60.58 ± 0.70	0.59 ± 0.03	16.47 ± 0.29	3.30 ± 0.11	3.30 ± 0.10	6.77 ± 0.15	2.23 ± 0.16	0.57 ± 0.04	1.31 ± 0.02	0.45 ± 0.034	5.32 ± 0.09	100.8
AND-6	28	58.78 ± 0.62	0.62 ± 0.02	14.78 ± 0.16	3.06 ± 0.11	3.46 ± 0.09	6.41 ± 0.11	2.20 ± 0.10	0.55 ± 0.04	1.52 ± 0.02	0.42 ± 0.012	6.85 ± 0.06	99.1
DCB-1	28	64.44 ± 0.52	1.03 ± 0.03	12.84 ± 0.16	3.61 ± 0.12	1.60 ± 0.05	3.93 ± 0.11	2.61 ± 0.15	1.17 ± 0.05	1.26 ± 0.04	0.39 ± 0.011	7.29 ± 0.08	100.2
DCB-2	47	62.17 ± 0.48	0.99 ± 0.03	12.38 ± 0.15	3.96 ± 0.10	1.55 ± 0.06	3.88 ± 0.09	2.83 ± 0.15	1.36 ± 0.07	1.38 ± 0.03	0.96 ± 0.009	8.58 ± 0.08	99.5
DCB-Al-1	29	58.27 ± 0.64	0.91 ± 0.03	20.46 ± 0.24	1.12 ± 0.07	1.51 ± 0.06	3.74 ± 0.11	3.01 ± 0.13	1.33 ± 0.07	0.76 ± 0.02	0.40 ± 0.012	9.18 ± 0.18	100.7
DCB-3	20	60.41 ± 0.57	0.96 ± 0.03	16.34 ± 0.21	1.33 ± 0.07	1.55 ± 0.06	4.13 ± 0.07	2.96 ± 0.15	1.28 ± 0.05	0.88 ± 0.02	0.44 ± 0.015	8.32 ± 0.10	99.0
DCB-4	25	58.51 ± 0.51	0.90 ± 0.02	15.83 ± 0.25	4.58 ± 0.13	1.34 ± 0.07	3.83 ± 0.09	3.18 ± 0.12	1.47 ± 0.07	1.10 ± 0.03	0.86 ± 0.058	8.01 ± 0.30	100.0
HSR-2	17	74.17 ± 0.54	0.14 ± 0.01	11.34 ± 0.15	0.81 ± 0.01	0.27 ± 0.02	0.98 ± 0.03	1.86 ± 0.11	2.30 ± 0.06	0.46 ± 0.02	0.45 ± 0.1085	7.56 ± 0.25	100.3
HSR-3	12	70.09 ± 0.83	0.15 ± 0.01	11.26 ± 0.23	0.80 ± 0.03	0.24 ± 0.01	1.00 ± 0.03	1.52 ± 0.08	2.34 ± 0.04	0.54 ± 0.03	0.49 ± 0.113	8.33 ± 0.32	97.2
AND-7	10	62.58 ± 0.47	0.63 ± 0.02	16.22 ± 0.17	1.25 ± 0.08	4.02 ± 0.23	6.51 ± 0.16	1.95 ± 0.14	0.52 ± 0.05	1.26 ± 0.03	0.22 ± 0.084	5.38 ± 0.12	100.7
DCB-5	15	66.14 ± 0.40	1.00 ± 0.03	17.69 ± 0.23	0.15 ± 0.05	1.51 ± 0.05	4.17 ± 0.08	2.87 ± 0.11	1.21 ± 0.06	0.75 ± 0.02	0.27 ± 0.0031	5.61 ± 0.31	101.5

Major elements and Cl were measured using EPMA (electron-probe microanalyzer), and H_2_O, and CO_2_ concentrations were measured using SIMS (secondary-ion mass spectrometry).

In order to integrate our data into a model of chlorine solubility, we started with the equation of Rusiecka and Wood (2025). In that paper we combined experimental data on hydrous basalt with 60 anhydrous results from Thomas and Wood (2021, 2023) and calculated chloride capacity (C_Cl_) for each experiment from:

2 $$\:{C}_{Cl}=\:\frac{Cl\:(wt.\:\%)}{\sqrt{f{Cl}_{2}}}\times\:\sqrt[4]{f{O}_{2}}$$

**Fig. 2:**
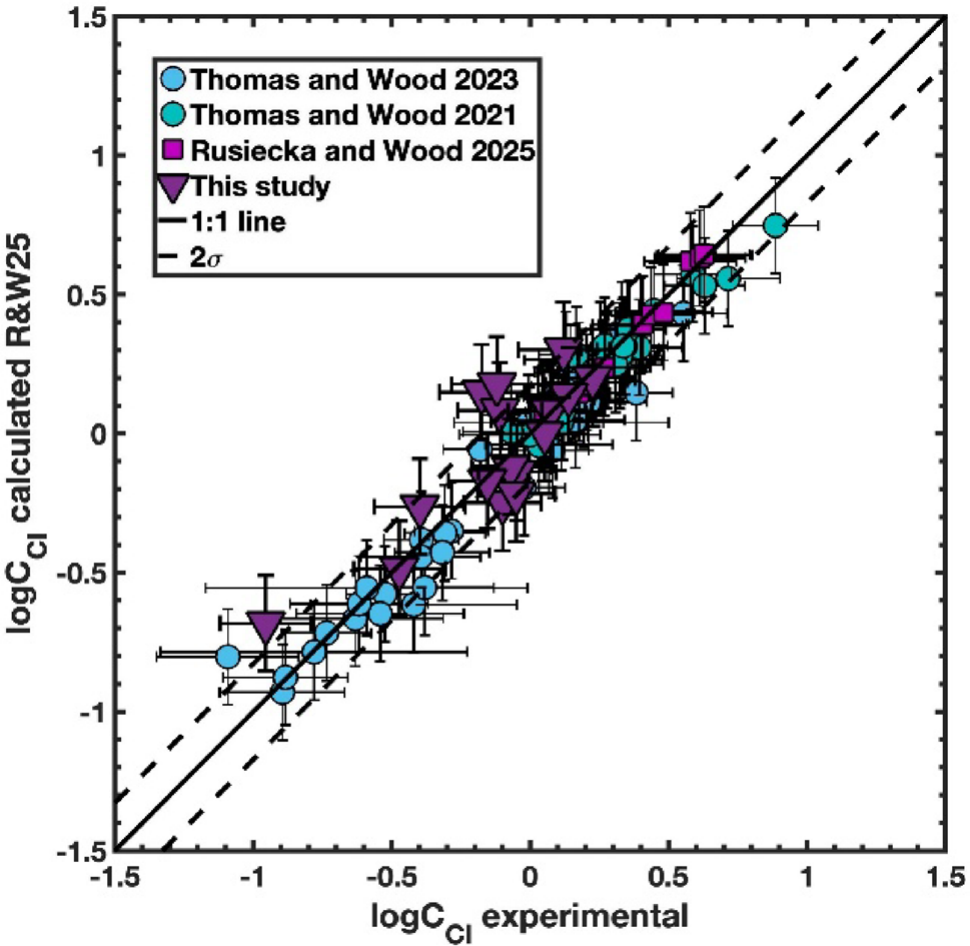
Logarithm of chloride capacity calculated using (Eq. 2, logC_Cl_ experimental) plotted versus calculated values from Eq. 3 (logC_Cl_ calculated) for the experiments of Thomas and Wood (2021, 2023), Rusiecka and Wood (2025), and this study. Solid line is 1:1 line, and the dashed lines are two standard errors of the fit to Eq. 3

We fitted the experimental logarithm of chloride capacity (Eq. 2) to mole fractions calculated on a single cation (hydrous) basis, including water as HO_0.5_, pressure, and temperature using SPSS software. We used stepwise linear regression applying the F-test (α = 0.05) at each step and excluding any terms that were not significant at α = 0.05. This produced the equation:

3 $$\:log{C}_{Cl}=1.492+\frac{4331{X}_{Ca}-3508{X}_{Si}+2440{X}_{Fe}-3921{X}_{K}-741P}{T}$$

with P in GPa, mole fractions on a single-cation basis (hydrous), standard error of 0.083, and R² = 0.963. This equation closely matches the Thomas and Wood (2023) anhydrous fit. Notably, the HO_0.5_ term was not significant, suggesting that water acts as an ideal diluent diluting the effects of other cations. Following a recent re-calibration of our piston-cylinder apparatus, we have made adjustments to the pressures used by Thomas and Wood (2021, 2023), and Rusiecka and Wood (2025). All corrected data (including data from Thomas and Wood 2021, 2023; Rusiecka and Wood 2025; and this study) are available in the Supplementary Material. For our experiments, we calculated chloride capacity using the definition of Eq. 2 and compared with the Rusiecka and Wood (2025) model of Eq. (3). As can be seen in Fig. 2, observed chloride capacities for the less silica-rich samples (basalts and andesites) are reproduced very well using the Rusiecka and Wood (2025) equation but this model slightly underestimates the value of logC_Cl_ (logarithm of chloride capacity) for more silica- (and crucially alkali-) rich melts.

We improved our model by refitting the full dataset, combining all existing measurements with the new hydrous data, using LASSO regression as implemented in the glmnet package (Friedman et al. 2010) in R (version 4.5.2). The fitting script was adapted from that used by Gorojovsky and Wood (2026). The penalty parameter (λ) was selected by Monte Carlo 10-fold cross-validation repeated 100 times. For each repeat, we chose the value of λ that lay within one standard error of the minimum mean squared error (MSE), and the average of these 100 λ values was then taken as the final penalty. The complete dataset was subsequently refit using LASSO with this final λ to obtain model coefficients and their uncertainties.

Uncertainties in the experimental logC_Cl_ values were calculated as standard errors, propagating assumed uncertainties of 0.1 log units in logfO₂, logfCl₂ (calculated using 0.05 GPa uncertainty on P, and 12 K on T) together with the standard deviation of measured chlorine contents for each experiment. All model inputs were standardised. Predictor variables included single-cation mole fractions (on a hydrous basis) of major oxides divided by temperature, pressure divided by temperature, and 1/T. The calibration data and the R script used for model fitting are openly available through the Oxford University Research Archive (ORA) DOI: 10.5287/ora-o8rnwkjr0. This procedure enables us to reassess the previous conclusion that HO_0.5_ can be treated as an ideal diluent (Rusiecka and Wood 2025).

Our new fit arrived at the equation:

4 $$\:log{C}_{Cl}=1.15+\frac{4359{X}_{Ca}-3055{X}_{Si}+2059{X}_{Fe}-3875{X}_{K}+163{X}_{Mg}-514P}{T}$$

with P in GPa, T in K, and mole fractions on a single-cation basis, standard error of 0.11, and R² = 0.94.

**Fig. 3:**
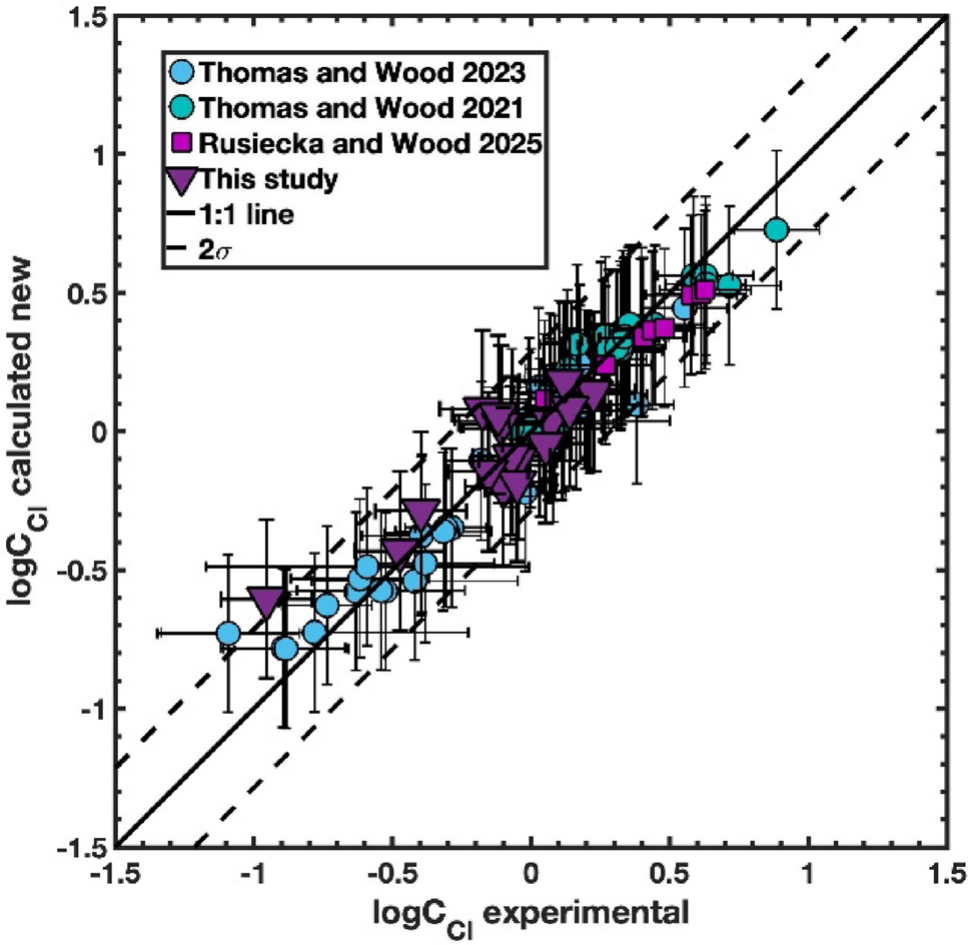
Logarithm of chloride capacity calculated using (Eq. 2, logC_Cl_ experimental) plotted versus calculated values from Eq. 4 (logC_Cl_ calculated) for the experiments of Thomas and Wood (2021, 2023), Rusiecka and Wood (2025), and this study. Solid line is 1:1 line, and the dashed lines are two standard errors of the fit to Eq. 4

Equation (4), while still correctly predicting chloride capacity in the hydrous basaltic melts of Rusiecka and Wood (2025) improves the prediction of logC_Cl_ for more silica (and alkali) rich samples (Fig. 3). What is most important, is that the term for HO_0.5_ was found not to be significant at the minimum level (α = 0.05). This means that the principal effect of water is that it dilutes the effects of the other cations on chloride capacity rather than itself having a strong impact on chlorine concentration even at water contents up to 8–9 wt%. The compositional dependence of the fitted chloride-capacity model indicates that Cl uptake is promoted by increasing Ca, Fe, and Mg contents and inhibited by increasing silica and K contents, so that chloride capacity rises systematically toward less silica-rich, less polymerized melt compositions; this behaviour is consistent with earlier experimental observations that Cl solubility increases with decreasing silica content (Webster et al. 2015; Thomas and Wood 2021, 2023).

### HCl fugacity and HCl content of volcanic gases

Hydrogen chloride (HCl) is the principal chlorine species in volcanic gases. Typical volcanic gases contain 0.1–10% HCl and HF, with only trace HBr and HI (Pyle and Mather 2009; Martin et al. 2012). Current global emissions are estimated at up to 5.5 Tg yr⁻¹ for HCl and 0.15–0.58 Tg yr⁻¹ for HF (Gerlach 2004; Aiuppa et al. 2009; Webster et al. 2018). These emissions affect both the atmosphere and the biosphere: Cl-bearing gases can transport metals such as Cu, Cd and Pb (Scholtysik and Canil 2021) and they contribute to stratospheric ozone loss through production of chlorine oxides ClO and OClO (von Glasow et al. 2009). In hydrothermal systems, halogens act as ligands and mobilise metals such as Cu and Au in ore-forming solutions (Blundy et al. 2015).

Despite its important environmental and economic impacts, the behaviour of chlorine during degassing is still not well understood, especially the link between melt composition and volatile budgets at depth on the one hand, and gas measurements at the surface on the other. Under nominally anhydrous conditions, Iwasaki and Katsura (1967) measured HCl solubility in silicate melts at 0.1 MPa and 1200–1290 °C, and derived separate melt–gas relations for rhyolite and basalt, but at unknown oxygen fugacity. No data are available for HCl solubility at higher pressures. By contrast, there is a large body of work on total Cl solubility in melts equilibrated with H₂O–NaCl(KCl) fluids. These studies show that Cl solubility (more precisely, the Cl content at Cl-rich aqueous fluid saturation) increases as melt polymerisation decreases and as the abundance of network-modifying cations rises (Webster et al. 2015). Furthermore, equilibrium Cl contents can reach several wt% in hydrous, depolymerised and peralkaline melts (e.g., Webster 1992; Carroll 2005). XANES and EXAFS studies on quenched melts (glasses) indicate influences of Ca, Mg, Fe and alkalis on the Cl bonding environment (Evans et al., 2008; Thomas et al. 2023), broadly consistent with the observed compositional dependence of chloride capacity (Thomas and Wood 2021, 2023; Rusiecka and Wood 2025).

Unlike NaCl, there are still very few experimental data that directly constrain the solubility of HCl in hydrous natural silicate melts at magmatic pressures. This distinction is important because HCl is favoured at low pressure, whereas NaCl becomes the dominant Cl-bearing species at higher pressure, as noted by Shinohara (2009).

The experiments of Thomas and Wood (2021) established that, in the absence of hydrogen, chlorine dissolves in silicate melts by replacing oxygen, as represented by the equilibrium:

5 $${\mathrm{Cl}}_{{\mathrm{2}}} {\text{ + [O}}^{{{\text{2 - }}}} {\text{] = 2[Cl}}^{ - } {\text{] + 0}}{\mathrm{.5O}}_{{\mathrm{2}}}$$

gas melt melt gas.

In the current study we have added known amounts of H_2_O to the melt, which generates significant fugacities of HCl (Table 2) at the known fugacities of oxygen, chlorine and H_2_O. As we have shown, the presence of up to 9 wt% H_2_O (corresponding to fHCl of ~ 2700 bars) has no discernible effect on chlorine solubility even at an fCl_2_/fHCl ratio of $\:{10}^{-8}$ (Table 2). Therefore, we can consider the chlorine dissolution reaction in the presence of hydrogen to be [5], the same as in the anhydrous system, indicating that Cl dissolves in the melt as Cl, not as HCl even in H_2_O-rich melts.

To calculate HCl fugacity in our hydrous Cl-bearing melts we use the two reactions:

6 $$\:\frac{1}{2}{H}_{2}+\frac{1}{2}{Cl}_{2}=HCl$$

7 $$\:{H}_{2}+\frac{1}{2}{O}_{2}={H}_{2}O$$

Equilibrium constants at 1 bar were obtained from the NIST- JANAF (https://janaf.nist.gov/, Chase 1998) Table

8 $$\:{K}_{{H}_{2}O}=\frac{{f}_{{H}_{2}O}}{{f}_{{H}_{2}}\times\:{f}_{{O}_{2}}^{0.5}}$$

9 $$\:{K}_{HCl}=\frac{{f}_{HCl}}{{f}_{{H}_{2}}^{0.5}\times\:{f}_{{Cl}_{2}}^{0.5}}$$

Using reaction 7, we can replace $\:{f}_{{H}_{2}}^{0.5}$ by $\:{\left(\frac{{f}_{{H}_{2}O}}{{K}_{{H}_{2}O}\times\:{f}_{{O}_{2}}^{0.5}}\right)}^{0.5}$ obtaining:

10 $$\:{K}_{HCl}=\frac{{f}_{HCl}}{\frac{{f}_{{H}_{2}O}^{0.5}}{{K}_{{H}_{2}O}^{0.5}\times\:{f}_{{O}_{2}}^{0.25}}\times\:{f}_{{Cl}_{2}}^{0.5}}$$

From there we can get the expression for $\:{f}_{HCl}$:

11 $$\:{f}_{HCl}={K}_{HCl}\times\:\frac{{f}_{{H}_{2}O}^{0.5}}{{K}_{{H}_{2O}}^{0.5}}\times\:\frac{{f}_{{Cl}_{2}}^{0.5}}{{f}_{{O}_{2}}^{0.25}}$$

From Eq. (2) we can replace $\:\frac{{f}_{{Cl}_{2}}^{0.5}}{{f}_{{O}_{2}}^{0.25}}$ in Eq. 11 by $\:\frac{Cl\:(wt.\:\%)}{{C}_{Cl}}$, eliminating the need to know $\:{f}_{{O}_{2}}$ and $\:{f}_{{Cl}_{2}}$. This yields f_HCl_ in terms of Cl content of the melt, chloride capacity C_Cl_ (from Eq. 4) and H_2_O fugacity, which comes from H_2_O content:

12 $$\:{f}_{HCl}={K}_{HCl}\times\:\frac{{f}_{{H}_{2}O}^{0.5}}{{K}_{{H}_{2O}}^{0.5}}\times\:\frac{Cl\:(wt.\:\%)}{{C}_{Cl}}$$

Where,

13 $$\:{\mathrm{log}}_{10}{K}_{{H}_{2}O}=\:\frac{12850}{T}-2.8675$$

and,

14 $$\:{\mathrm{log}}_{10}{K}_{HCl}=\:\frac{4894.6}{T}+0.3436$$

Both Eq. (13) and Eq. (14) are valid between 300 and 3000 K.

Several models have been developed to describe chlorine behaviour during magmatic degassing. Approaches such as SolEx (Witham et al. 2012) and related models treat chlorine through melt–fluid partitioning relationships, typically using experimentally calibrated distribution coefficients. More recent thermodynamic frameworks, such as D-Compress (Burgisser et al. 2015; Alletti et al. 2014), combine equilibrium gas speciation with mass-balance constraints, but rely on empirical solubility relationships linking melt chlorine content to HCl fugacity. In these models, fluid compositions are not measured directly but reconstructed from mass balance, often under conditions where low fluid/melt ratios are required to minimise dissolution of melt components into the fluid. This can lead to large uncertainties in calculated fluid compositions, which propagate into the parameterisation of chlorine solubility.

In contrast, our approach does not rely on empirical solubility laws or reconstruction of fluid composition. HCl fugacity is instead derived directly from equilibrium relationships involving H₂–H₂O–HCl and the experimentally constrained chloride capacity (C_Cl_), allowing gas composition to be calculated solely from melt composition, temperature, pressure and fH₂O. The disadvantage is that it makes no provision for metal-chloride species in the fluid and is only applicable to low salinity fluids. However, because this formulation is based on fundamental thermodynamic relationships, it is independent of any particular degassing framework and can be implemented within existing models such as SolEx or D-Compress to improve their treatment of chlorine speciation.

We developed a C–H–O–S–Cl speciation code for fluids in equilibrium with silicate melts to calculate fugacities of HCl from melt composition and to account for other volatile species during decompression and fluid release. Water fugacity was obtained from water content of the melt using either the Burnham (1994) or Moore (1998) relations, depending on pressure (Moore (1998) equation is calibrated only up to 300 MPa therefore for higher pressures we used the Burnham (1994) model). Carbon content and speciation in the melt was adopted from Eguchi and Dasgupta (2018), while sulphur content and speciation in the melt follows Gorojovsky and Wood (2026). The oxidation state of iron was calculated from Kress and Carmichael (1991), and HCl/Cl_2_​ fugacities follow the equations derived in this study. All equations used are presented in the Supplementary material. Imperfect behaviour of the pure gases is treated with the modified Redlich–Kwong (MRK) equation of state (Holloway 1977) using Van der Waals mixing rules. Critical properties used to calculate corresponding MRK parameters were taken from Reid et al. (1987). In our approach all Cl in the gas is calculated as HCl, and no other Cl-bearing species are considered. Although not reasonable at high pressures where salt-forming chlorides (mainly NaCl) tend to partition into an aqueous fluid, at low pressure (< 50 MPa), NaCl exsolution becomes insignificant and most chlorine is lost as HCl (Shinohara 2009).

**Fig. 4:**
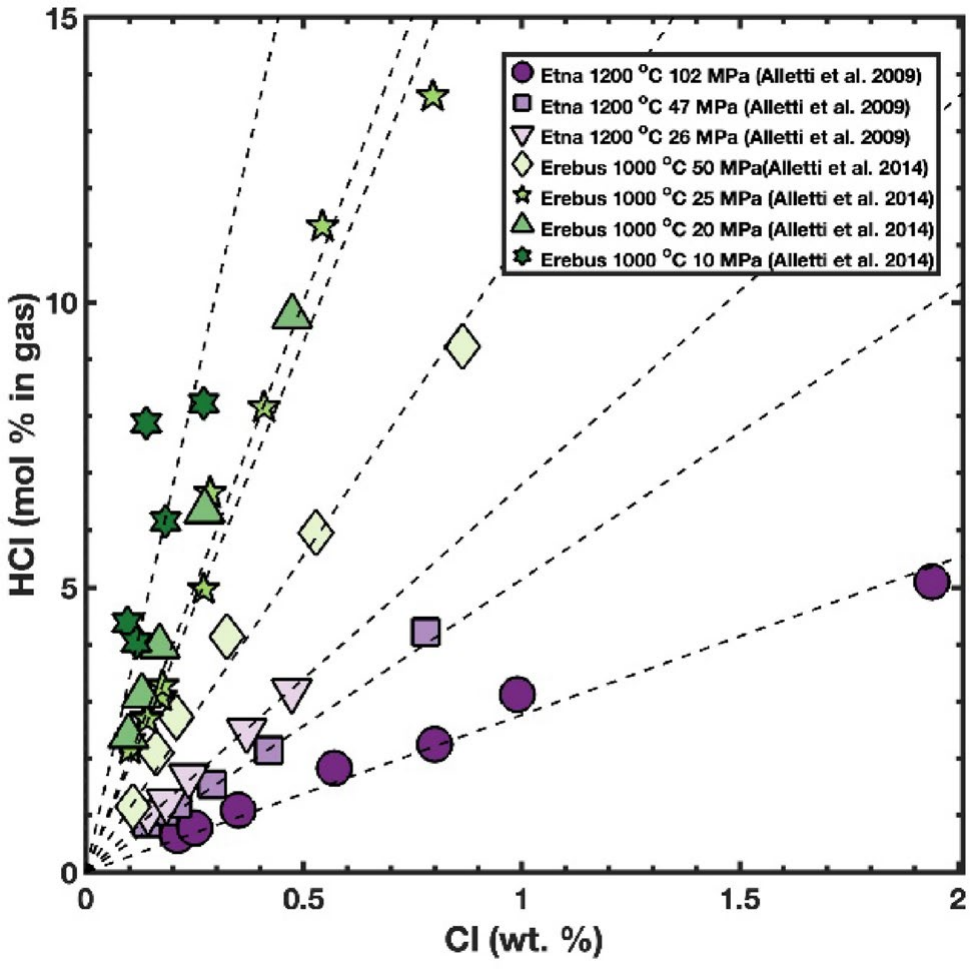
Cl in the melt (wt%) versus HCl content of the gas phase (mol %) calculated with Eq. 11 and adjusted with the MRK equation of state. Data from selected experiments saturated in H_2_O-rich fluid: Alletti et al. (2009, 2014)

We calculated HCl fugacities and HCl contents of gases in equilibrium with melts in selected experiments from the literature. We chose two contrasting compositions – basalt from Mt. Etna (Alletti et al. 2009), and phonolite from Mt. Erebus (Alletti et al. 2014). At low pressure and near H₂O saturation, HCl in the fluid phase scales linearly with Cl dissolved in the melt; the line passes through the origin and the slope varies with conditions (mainly pressure) and melt composition (Fig. 4). As chlorine (and therefore HCl) is more soluble in basalt than in phonolite the gradients of the lines are lower for basalt than for phonolite. HCl contents of the gas increase with decreasing pressure because of the decreasing solubility of H_2_O in the melt as pressure decreases.

The model used for these calculations is available online (https://github.com/mkrusiecka/HCl_fugacity). Given pressure, temperature, and melt composition, it returns gas speciation in equilibrium with any melt and is most applicable to melt inclusion data such as those discussed in the following section.

HCl and Cl_2_ in volcanic systems – insights from melt inclusions We compiled melt inclusion data from a number of volcanoes worldwide (Piton de la Fournaise, Réunion, Bureau et al. 1998; La Soufrière, St. Vincent, Metcalfe et al. 2021; Ambrym, Vanuatu, Allard et al. 2016; Etna 2002–2003 eruption, Spilliaert et al., 2006; Pinatubo 1991 eruption, Borisova et al. 2005; Pico, Azores, Métrich et al. 2014; Unzen 1991–1995, Botcharnikov et al. 2008; Popocatépetl, Witter et al. 2005; Masaya, Zurek et al. 2019) and calculated chloride capacity, chlorine fugacity, HCl fugacity, and SO_2_/HCl ratios in the coexisting gases from the compositions of the melts. Data were selected to represent a wide compositional range and on the basis that estimates of temperature and oxygen fugacity were available.

**Fig. 5:**
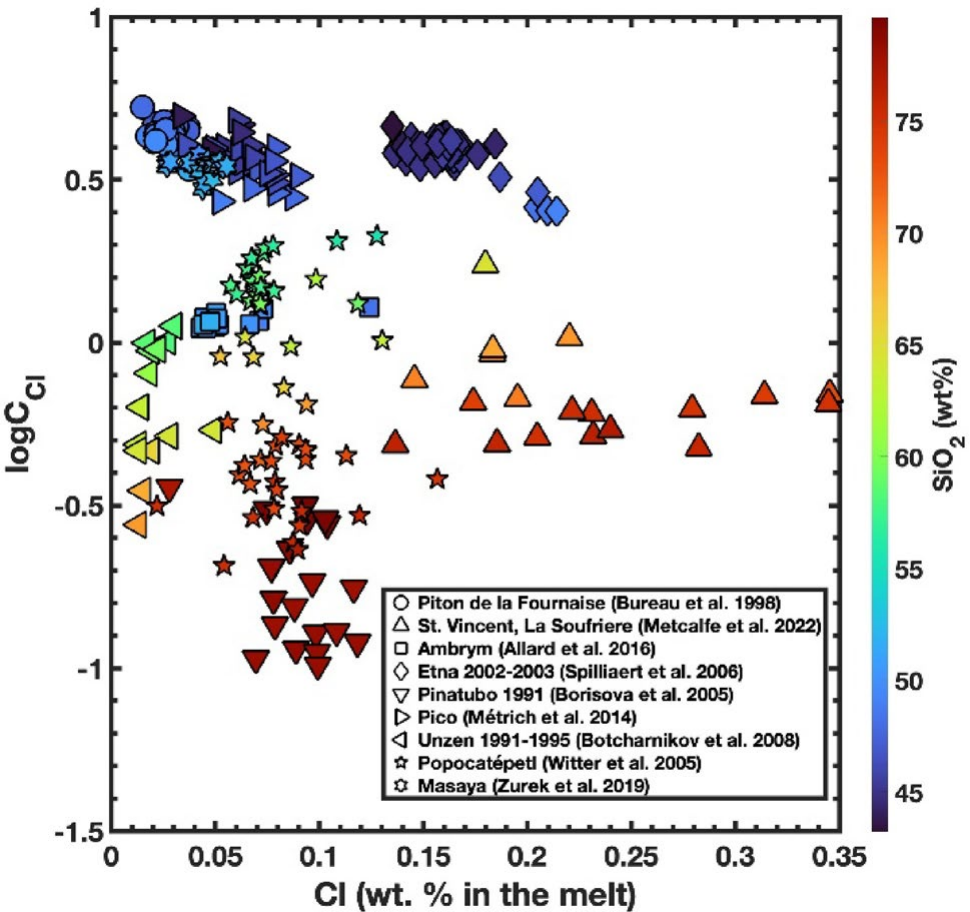
Variation of chloride capacity and chlorine content of melt inclusions from different volcanoes worldwide. Cl content (wt%) is plotted on x-axis, logC_Cl_ on y-axis, and silica content (in wt%) defines the colour scale

Strictly speaking, solubility is the maximum amount of a component that can dissolve in a solvent to form a stable solution. In this context, chlorine solubility should be the maximum concentration of Cl that a silicate melt can hold while in equilibrium with pure Cl₂ at a specified temperature and pressure. This definition is, however, not specific enough and chloride capacity (C_Cl_) refines it by accounting for the dependence of chlorine content on temperature (T), pressure (P), the fugacities of chlorine (fCl₂) and oxygen (fO₂), and melt composition. Because the gas coexisting with terrestrial melts is never 100% Cl₂, melts are never—strictly speaking “chlorine saturated.” Nevertheless, C_Cl_ provides chlorine content under given P–T–fO₂–fCl₂ conditions. There have been several detailed studies of the solubility and behaviour of chlorine in hydrous silicate melts (e.g., Webster 1992; Webster et al. 1999, 2015; Signorelli and Carroll, 2002; Carroll 2005; Stelling et al. 2008; Alletti et al. 2009; Alletti et al. 2014). These works are commonly referred to as studies of “chlorine solubility”, but in practice they investigate silicate melts in equilibrium with Cl-rich (mostly NaCl-bearing) aqueous fluids. They are therefore best viewed as constraints on chlorine contents at brine (or Cl-rich aqueous fluid) saturation, analogous to SCSS (sulphur content at sulphide saturation). Figure 5 is a graph of log₁₀C_Cl_ versus Cl contents of melt inclusions. For more silica-rich compositions, log₁₀C_Cl_ typically ranges from − 1 to 0 (C_Cl_ ≈ 0.1–1), whereas the chloride capacities of basaltic melts are generally 1 to 2 orders of magnitude higher, log₁₀C_Cl_ ≈ 0 to 1 (C_Cl_ ≈ 1–10). In contrast, silica-rich melts tend to have higher Cl contents than basaltic melts, this difference reflecting the fact that Cl behaves incompatibly, concentrating in the melt during magmatic differentiation.

**Fig. 6:**
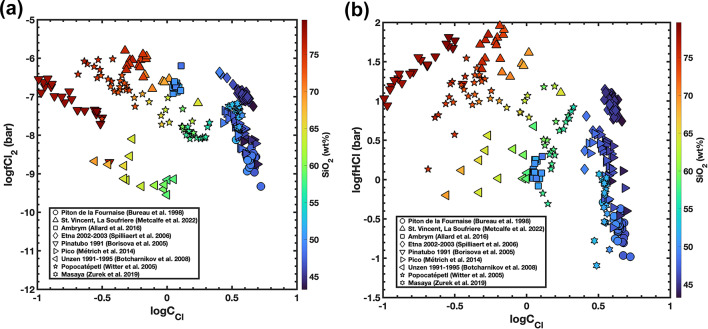
Variation of chloride capacity and chlorine (**a**) and HCl (**b**) fugacities in equilibrium with melt inclusions from different volcanoes worldwide. logC_Cl_ is plotted on the x-axis, logfCl_2_ (6a) and logfHCl (6b) on the y-axis, and silica content (in wt%) defines the colour scale

Figure 6 shows the variation of fugacities of chlorine species in the melt-inclusion compilation: fCl₂ generally lies around 10⁻¹⁰ to 10⁻^6^ bar (6a), while fHCl, is 5 or more orders of magnitude higher at approximately 10⁻¹ to 10² bar (6b). This explains the dominance of HCl in chlorine species emitted by volcanoes.

**Fig. 7:**
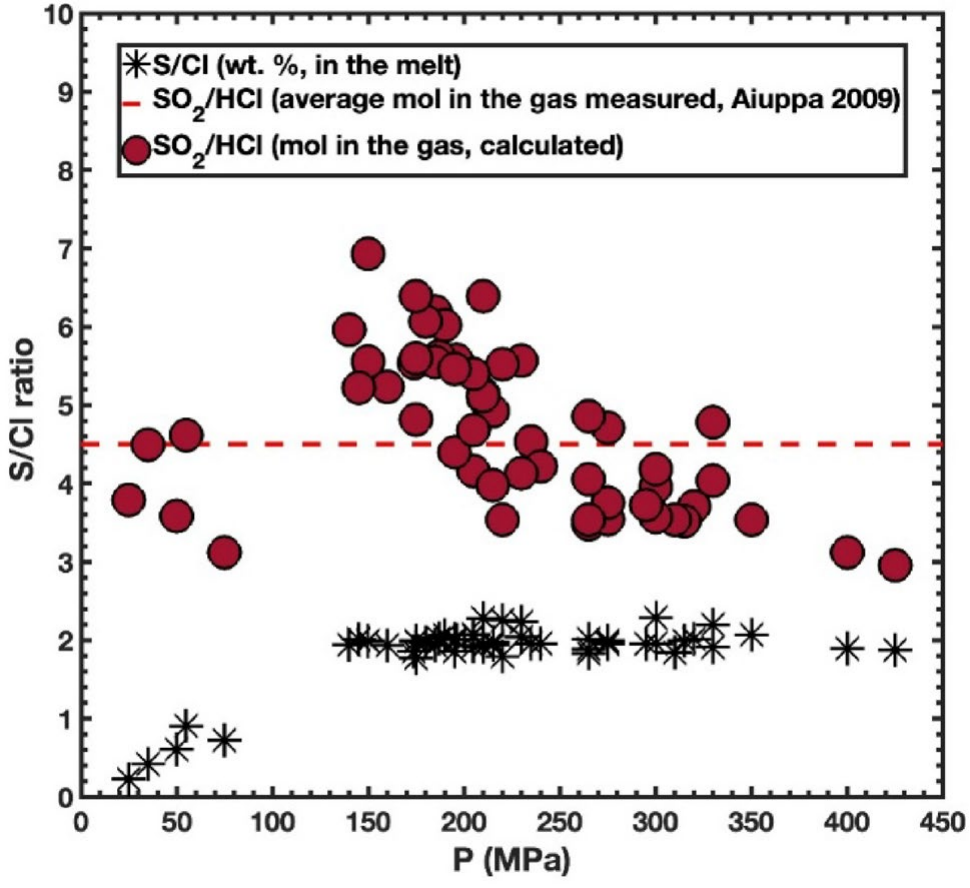
Variation of S/Cl (in wt%) ratio in melt inclusions from 2002–2003 eruption of Mt. Etna (Spilliaert et al. 2006) and SO_2_/HCl ratio in the fluid phase in equilibrium with the melt inclusions (in mol, calculated using C-H-O-S-Cl MRK EOS)

We applied our gas composition model (using Moore’s model for H_2_O fugacity) to melt inclusions from the 2002–2003 Etna eruption (Spilliaert et al. 2006). The basalts produced in this highly explosive eruption were amongst the most primitive ones of the last 300 years and are very rich in volatile elements with an average H_2_O content of 3.4 ± 0.2 wt% (Spilliaert et al. 2006). We assumed them to be volatile-saturated and followed Spilliaert et al. (2006) to calculate pressure using VolatileCalc (Newman & Lowenstern, 2002). We set T = 1160 °C and logfO₂ close to the nickel – nickel oxide buffer ($\:{\Delta\:}NNO=\:-0.4$; Gennnaro et al. (2020) cited a range of fO_2_ of NNO − 0.9 to + 0.4 for Etna basalts, including these from the 2002–2003 eruption). For each inclusion we computed gas speciation and the SO₂/HCl ratio and also total S_tot_/HCl. Halogen concentrations in volcanic plumes vary widely in space and time because they depend on the degree of mixing between halogen-free air and volcanic gases. To minimise the dilution effect, gas geochemists routinely normalise HCl (and HF) to SO₂ in plume samples and fumarole condensates, reporting SO₂/HCl (and SO₂/HF) ratios. These ratios are largely independent of plume dilution and, in short-range measurements of quiescent, ash-free plumes, have been observed to show no systematic change with plume aging, suggesting only limited atmospheric processing over these short transport times. (e.g., Aiuppa et al. 2007). The average SO₂/HCl which we calculate is ~ 4.6 (Fig. 7), matching the mean value measured in volcanic gases during the eruption (~ 4.5; Aiuppa et al. 2004). SO₂/HCl remains stable at high pressure (between 300 and 250 MPa), then increase, finally dropping slightly at pressures below 50 MPa (Fig. 7).

Figure 7 also shows the weight ratio of S to Cl in the melt, which stays constant until about 140 MPa and then begins to fall. This behaviour is due principally to the differences in pressure dependence of sulphate (and sulphide) and Cl capacities. Both S and Cl remain in the melt until the onset of rapid S degassing at around 140 MPa, which is reflected in the changing SO_2_/HCl ratio in the gas phase (and S/Cl ratio in the melt). The ability to compute SO₂/HCl and S/HCl directly from melt inclusions also provides a quantitative framework for evaluating excess degassing and magma plumbing. Volatile ratios inferred at inclusion entrapment pressures can be compared with plume SO₂/HCl measured at the surface, yielding estimates of open-system gas loss and gas–melt decoupling along the ascent path. For open-conduit systems such as Etna, time-dependent changes in SO₂/HCl predicted by our model as magma ascends, ponds, or mixes can be compared with gas-monitoring data, linking subsurface storage and recharge processes to observed variations in plume composition that are used in eruption forecasting.

**Fig. 8:**
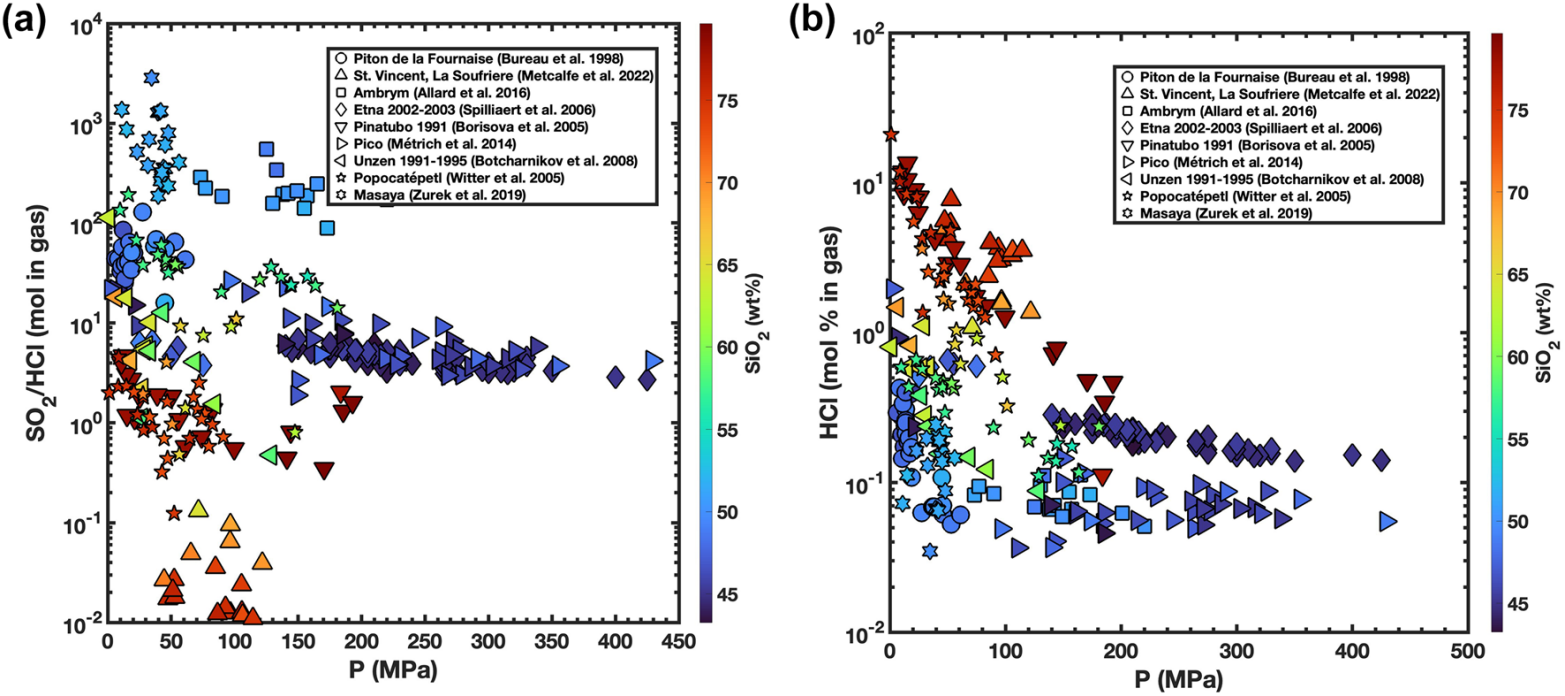
Pressure (MPa) is plotted on x-axis, SO_2_/HCl (8a), and HCl content (8b, mol %) in the gas in equilibrium with melt inclusions on y-axis, and silica content (in wt%) defines the colour scale

We calculated the SO_2_/HCl ratio for the gases in equilibrium with all the melt inclusions from our worldwide compilation. This indicates that the ratio in basalts typically varies between 5 and 100, while in more silica-rich compositions it is typically 10^− 2^ to 10^− 1^ (Fig. 8a). These ratios correspond to calculated HCl content of the gas of 10^− 2^ to 1 mol % in equilibrium with basalts and 1 to 10 mol % HCl in equilibrium with more silica rich compositions (Fig. 8b).

### Formation of reactive halogen species in volcanic plumes

Volcanic chlorine can influence atmospheric chemistry not only because volcanoes emit abundant HCl, but also because processes at the vent and in the near-vent plume can convert this chlorine into reactive forms. Martin et al. (2006) proposed that vents act as high-temperature reaction sites where small amounts of ambient air mix with hot magmatic gases, and that even a few percent air in an H_2_O-rich volcanic gas can generate elevated abundances of reactive halogen species in the plume. In their thermodynamic framework, the addition of atmospheric oxygen promotes oxidative dissociation of HCl (and HBr), releasing halogen radicals in the high-temperature mixture; as the plume cools, these radicals are then further oxidized (e.g., by OH) to form detectable halogen oxides such as ClO (Martin et al. 2006). They noted explicitly that low-temperature oxidation of Cl produced by high-temperature dissociation of HCl could account for the tens-of-ppbv ClO observed downwind in volcanic plumes (e.g., Sakurajima; Martin et al. 2006). Because ClO (and OClO) participates in catalytic ozone-loss cycles, this vent-to-plume reaction pathway makes volcanic chlorine potentially important for ozone chemistry in the troposphere in the vicinity of volcanic plumes (von Glasow et al. 2009). On a per atom basis, bromine is even more effective at consuming ozone than chlorine (e.g., Klobas et al. 2020) but its lower abundance means that it is not quantitatively more environmentally significant.

**Fig. 9:**
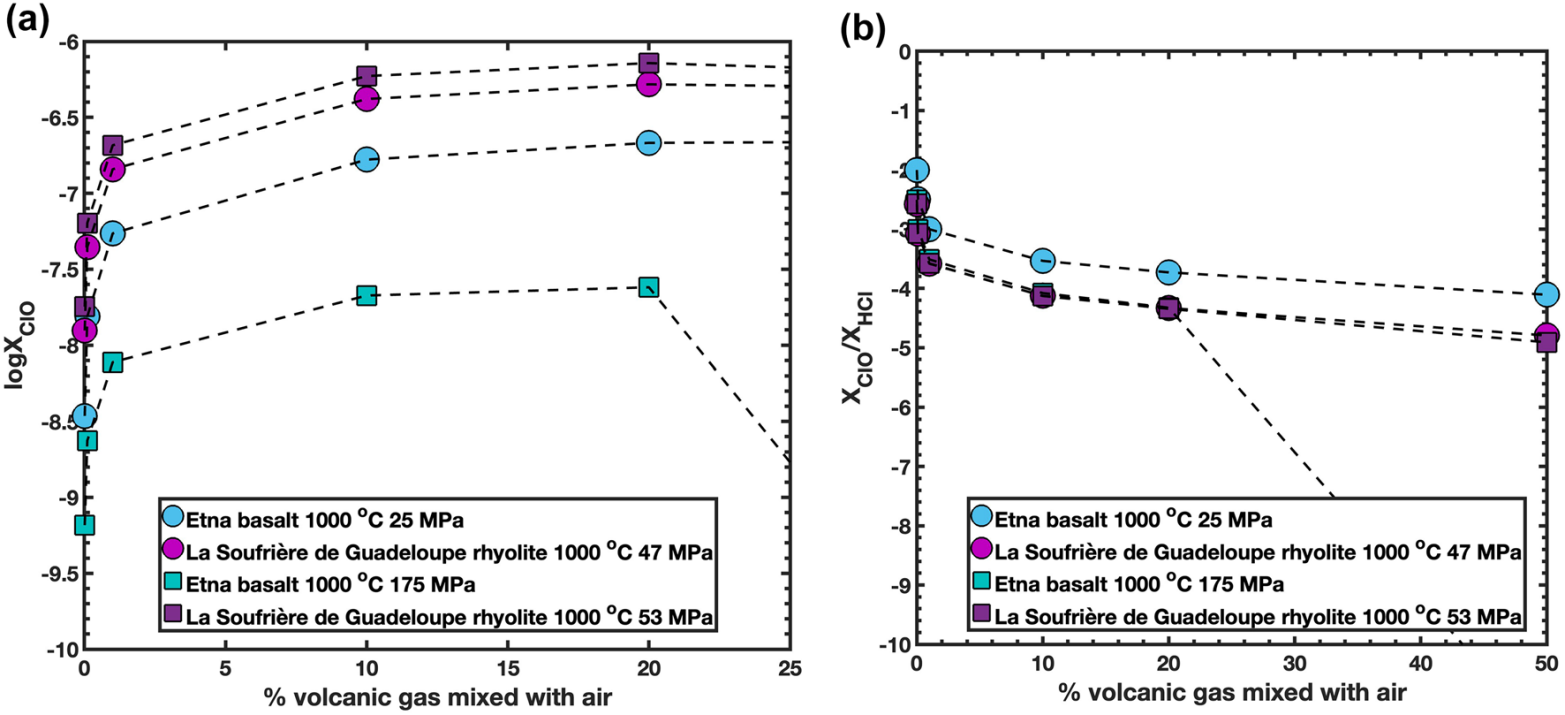
**a** logX_ClO_ (logarithm of mol fraction of ClO produced) plotted against percent of volcanic gas mixed with air at 1000 °C. X_ClO_ was calculated from composition of gas in equilibrium with melt inclusions from Etna (basalt), and La Soufrière (rhyolite) using HSC Chemistry GEM Equilibrium Compositions Module. **b **ratio of X_ClO_/X_HCl_ in the gas against % of volcanic gas mixed with air calculated using the same conditions and compositions as a)

To explore these processes quantitatively, we used two contrasting melt inclusion compositions (La Soufrière, St. Vincent; Metcalfe et al. 2021; and the 2002–2003 Etna eruption; Spilliaert et al., 2006) and calculated ClO production from volcanic gases mixed with air in different proportions using the HSC Chemistry GEM Equilibrium Compositions Module. We mixed 0.01, 0.1, 1, 10, 20, and 50% volcanic gas with air at 1000 °C, using gas compositions in equilibrium with basaltic and rhyolitic melt inclusions at two pressures (25 and 175 MPa for Etna, and 47 and 53 MPa for La Soufrière). Figure 9a shows logX_ClO_ (logarithm of the mol fraction of ClO) plotted against the percentage of volcanic gas mixed with air: as the volcanic gas fraction increases to 10%, X_ClO_ rises steeply and then stabilizes. When gas in equilibrium with the Etna basalt composition at 175 MPa is mixed with air in a 50/50 proportion, however, ClO production drops sharply because increased water content of the gas mixture promotes oxidation and depletes HCl in the gas. Figure 9b shows the ratio X_ClO_/X_HCl_ against the percentage of volcanic gas mixed with air; this ratio is relatively stable but is influenced by the proportions of species other than chlorine and HCl, mainly H_2_O and CO_2_. For Etna at 25 MPa, the ratio is two orders of magnitude lower than for rhyolite because the gas in equilibrium with this composition is mostly CO_2_, whereas the other compositions are dominantly water-rich. Gas compositions mixed with air in different proportions, along with a list of all species used for the modelling, are provided in the Supplementary Material.

## Conclusions

We performed experiments on hydrous andesite, dacite and rhyolite melts at 1200 °C and 0.5–1.63 GPa, with buffered fugacities of chlorine and oxygen, in order to determine the effect of H_2_O on chloride capacities C_Cl_. When the results are added to available data on anhydrous melts and regressed against appropriate compositional terms, we find that, even with ~ 8–9 wt% H_2_O, the explicit hydrogen term (HO_0.5_) is not statistically significant in the regression. The dominant influence of H_2_O on melt chloride capacity is therefore simple dilution of the effects of other network-forming/modifying cations rather than specific H–Cl interactions.

Accordingly, we revised our chloride-capacity model to improve the fit for evolved melts. Refitting the full data set (anhydrous plus new hydrous runs) with updated pressures and temperatures gave:

$$\:log{C}_{Cl}=1.15+\frac{4359{X}_{Ca}-3055{X}_{Si}+2059{X}_{Fe}-3875{X}_{K}+163{X}_{Mg}-514P}{T}$$

with P in GPa, T in K, and mole fractions on a single-cation basis.

Combining chloride capacity with thermodynamic data for equilibria involving HCl and H_2_O yields:

$$\:{f}_{HCl}={K}_{HCl}\times\:\frac{{f}_{{H}_{2}O}^{0.5}}{{K}_{{H}_{2O}}^{0.5}}\times\:\frac{Cl\:(wt.\:\%)}{{C}_{Cl}}$$

This allows direct estimation of $\:{f}_{\mathrm{H}\mathrm{C}\mathrm{l}}$ from melt composition, $\:T$, $\:P$, and $\:{f}_{{\mathrm{H}}_{2}\mathrm{O}}$—without explicit knowledge of $\:{f}_{\mathrm{C}{\mathrm{l}}_{2}}$ or $\:{f}_{{\mathrm{O}}_{2}}$.

At low pressure and near H_2_O saturation, HCl content of the gas scales linearly with Cl dissolved in the melt and trends pass through the origin; the slopes vary systematically with melt composition and pressure-temperature conditions. This behaviour provides a simple diagnostic for back-calculating gas composition from melt-inclusion Cl content.

When this approach is applied to published melt-inclusion data sets, typical $\:{f}_{\mathrm{C}{\mathrm{l}}_{2}}$ values are ~ 10^− 10^ to 10^− 6^ bar, while $\:{f}_{\mathrm{H}\mathrm{C}\mathrm{l}}$ is several orders of magnitude higher (~ 10^− 1.5^ to 10^2^ bar), thus explaining why Cl_2_ is generally at very low concentration in volcanic plumes while HCl is abundant. The modelled SO_2_/HCl ratio for Etna 2002–2003 (~ 4.6) matches measurements of emitted gases, further demonstrating the validity of our approach.

Melt S/Cl and gas SO_2_/HCl evolve with decompression in ways captured by the model and consistent with observations. In combination with melt-inclusion datasets, the model therefore provides a route from petrological constraints on Cl and H_2_O contents to quantitative estimates of magmatic HCl fluxes to the atmosphere and oceans. These fluxes can be used as inputs for regional and global models of halogen impacts on atmospheric composition and climate.

Volcanic HCl is not atmospherically inert: high-temperature air–gas mixing activates HCl into low abundance but highly reactive chlorine radicals. Following Martin et al. (2006), limited entrainment of air into hot magmatic gas promotes oxidative dissociation of HCl to produce reactive Cl, which is then oxidized during plume cooling to form ClO (and potentially OClO), resulting in reduction of ozone concentration. To evaluate this activation potential in our own system, we modelled equilibrium ClO production (HSC Chemistry GEM) by mixing volcanic gases—derived from contrasting melt inclusion compositions for La Soufrière and Etna at relevant pressures—with air over a wide range of proportions at 1000 °C. These calculations show that ClO production rises rapidly at low degrees of air addition and then stabilizes with further mixing, while becoming limited where the available HCl in the gas is insufficient (e.g., for the Etna basalt case at 175 MPa under 50/50 mixing). The ratio X_ClO_/X_HCl_ remains relatively stable but varies systematically with bulk gas composition, particularly the H_2_O/CO_2_ ratio, demonstrating the importance of other major components to the efficiency of generation of ClO and ozone loss. Together, this approach provides a direct bridge from melt-based constraints on $\:{f}_{\mathrm{H}\mathrm{C}\mathrm{l}}$ and HCl fluxes to the formation of reactive chlorine in volcanic plumes and motivates coupling the present petrological framework to plume-chemistry models when assessing downwind atmospheric impacts.

A key simplification in the present implementation is the assumption that all chlorine in the gas phase is present as HCl. This approximation is appropriate at low pressure (< 50 MPa) and high H_2_O fugacity, where experiments and volcanic gas measurements indicate that HCl is the dominant Cl-bearing species in the vapour phase. At higher pressures and salinities, however, a significant fraction of Cl is expected to partition into NaCl- and KCl-bearing aqueous fluids or brines, and neglecting these species will underestimate the total Cl budget and modify the relationship between $\:{C}_{\mathrm{C}\mathrm{l}}$and $\:{f}_{\mathrm{H}\mathrm{C}\mathrm{l}}$. Extending the C–H–O–S–Cl speciation code to include an explicit NaCl (and KCl) component will be essential for treating brine-rich, porphyry-style systems and for quantitatively modelling halogen partitioning at higher pressures.

## Supplementary Information

Below is the link to the electronic supplementary material.

Supplementary Material 1

Supplementary Material 2

## Data Availability

The data that support the findings of this study, and R script used to fit Eq. 4 are openly available through the Oxford University Research Archive (ORA) DOI: 10.5287/ora-o8rnwkjr0.

## References

[CR3] Aiuppa A, Baker DR, Webster JD (2009) Halogens in volcanic systems. Chem Geol 263:1–18. 10.1016/j.chemgeo.2008.10.005

[CR1] Aiuppa A, Federico C, Giudice G, Gurrieri S, Paonita A, Valenza M (2004) Plume chemistry provides insights into mechanisms of sulfur and halogen degassing in basaltic volcanoes. Earth Planet Sci Lett 222:469–483. 10.1016/j.epsl.2004.03.020

[CR2] Aiuppa A, Franco A, von Glasow R, Allen AG, D’Alessandro W, Mather TA, Pyle DM, Valenza M (2007) The tropospheric processing of acidic gases and hydrogen sulphide in volcanic gas plumes as inferred from field and model investigations. Atmos Chem Phys 7:1441–1450. 10.5194/acp-7-1441-2007

[CR4] Allard P, Burton M, Sawyer G, Bani P (2016) Degassing dynamics of basaltic lava lake at a top-ranking volatile emitter: Ambrym volcano, Vanuatu arc. Earth Planet Sci Lett 448:69–80. 10.1016/j.epsl.2016.05.014

[CR5] Alletti M, Baker DR, Scaillet B, Aiuppa A, Moretti R, Ottolini L (2009) Chlorine partitioning between a basaltic melt and H_2_O-CO_2_ fluids at Mount Etna. Chem Geol 263:37–50. 10.1016/j.chemgeo.2009.04.003

[CR6] Alletti M, Burgisser A, Scaillet B, Oppenheimer C (2014) Chloride partitioning and solubility in hydrous phonolites from Erebus volcano: A contribution towards a multi-component degassing model. GeoResJ. 10.1016/j.grj.2014.09.003

[CR7] Blundy J, Mavrogenes J, Tattitch B, Sparks S, Gilmer A (2015) Generation of porphyry copper deposits by gas-brine reaction in volcanic arcs. Nat Geosci 8:235–240. 10.1038/ngeo2351

[CR8] Borisova AY, Pichavant M, Beny JM, Rouer O, Pronost J (2005) Constraints on dacite magma degassing and regime of the June 15, 1991, climactic eruption of Mount Pinatubo (Philippines): new data on melt and crystal inclusions in quartz. J Volcanol Geotherm Res 145:35–67. 10.1016/j.jvolgeores.2005.01.004

[CR9] Botcharnikov RE, Holtz F, Almeev RR, Sato H, Behrens H (2008) Storage conditions and evolution of andesitic magma prior to the 1991-95 eruption of Unzen volcano: constraints from natural samples and phase equilibria experiments. J Volcanol Geotherm Res 175:168–180. 10.1016/j.jvolgeores.2008.03.026

[CR10] Boulliung J, Wood BJ (2022) SO_2_ solubility and degassing behavior in silicate melts. Geochim Cosmochim Acta 336:150–164. 10.1016/j.gca.2022.08.032

[CR11] Boulliung J, Wood BJ (2023) Sulfur oxidation state and solubility in silicate melts. Contrib Mineral Petrol 178:56. 10.1007/s00410-023-02033-9

[CR13] Brooker RA, Kohn SC, Holloway JR, McMillan PF (2001) Structural controls on the solubility of CO_2_ in silicate melts: part I: bulk solubility data. Chem Geol 174:225–239. 10.1016/S0009-2541(00)00353-3

[CR12] Brooker RA, Kohn SC, Holloway JR, McMillan PF, Carroll MR (1999) Solubility, speciation and dissolution mechanisms for CO_2_ in melts on the NaAlO2-SiO2 join. Geochim Cosmochim Acta 63:3549–3565. 10.1016/S0016-7037(99)00196-9

[CR14] Bureau H, Pineau F, Métrich N, Semet MP, Javoy M (1998) A melt and fluid inclusion study of the gas phase at Piton de la Fournaise volcano (Réunion Island). Chem Geol 147:115–130. 10.1016/S0009-2541(97)00176-9

[CR15] Burgisser A, Alletti M, Scaillet B (2015) Simulating the behavior of volatiles belonging to the C-O-H-S system in silicate melts under magmatic conditions with the software D-Compress. Comput Geosci 79:1–14. 10.1016/j.cageo.2015.03.002

[CR16] Burnham CW (1994) Development of the Burnham model for prediction of H_2_O solubility in magmas.Volatiles in magmas. Rev Miner 30:123–129

[CR17] Candela PA, Holland HD (1986) A mass transfer model for copper and molybdenum in magmatic hydrothermal systems; the origin of porphyry-type ore deposits. Econ Geol 81:1–19. 10.2113/gsecongeo.81.1.1

[CR18] Carmichael IS, Turner FJ, Verhoogen J (1974) Igneous petrology. McGraw-Hill, New York

[CR19] Carroll MR (2005) Chlorine solubility in evolved alkaline magmas. Ann Geophys 48:4–5

[CR20] Chase M (1998) NIST-JANAF Thermochemical Tables, 4th edn. American Institute of Physics

[CR21] de Ronde CEJ, Humphris SE, Höfig TW (2019) Expedition 376 Scientists Brothers arc flux. Proceedings of the International Ocean Discovery Program 376. International Ocean Discovery Program, College Station, TX. 10.14379/iodp.proc.376.2019

[CR22] De Vivo B, Lima A, Webster JD (2005) Volatiles in magmatic-volcanic systems. Elements 1:19–24. 10.2113/gselements.1.1.19

[CR23] Ding S, Plank T, Wallace PJ, Rasmussen DJ (2023) Sulfur_X: A model of sulfur degassing during magma ascent. Geochem Geophys Geosyst 24:e2022GC010552. 10.1029/2022GC010552

[CR24] Eguchi J, Dasgupta R (2018) A CO_2_ solubility model for silicate melts from fluid saturation to graphite or diamond saturation. Chem Geol 487:23–38. 10.1016/j.chemgeo.2018.04.012

[CR78] Evans, K. A., J. A.Mavrogenes, H. S.O'Neill, N. S.Keller, and L.-Y.Jang (2008), A preliminary investigation of chlorine XANES in silicate glasses, Geochem Geophys Geosyst, 9, Q10003 10.1029/2008GC002157

[CR25] Filiberto J, Treiman AH (2009) The effect of chlorine on the liquidus of basalt: first results and implications for basalt genesis on Mars and Earth. Chem Geol 263:60–68. 10.1016/j.chemgeo.2008.08.025

[CR26] Fincham CJB, Richardson FD (1954) The behaviour of sulphur in silicate and aluminate melts. Proc R Soc Lond Ser A Math Phys Sci 223:40–62. 10.1098/rspa.1954.0099

[CR27] Friedman JH, Hastie T, Tibshirani R (2010) Regularization paths for generalized linear models via coordinate descent. J Stat Softw 33:1–22. 10.18637/jss.v033.i01 20808728 PMC2929880

[CR28] Gennaro E, Paonita A, Iacono-Marziano G, Moussallam Y, Pichavant M, Peters N, Martel C (2020) Sulphur behaviour and redox conditions in Etnean magmas during magma differentiation and degassing. J Petrol 61(10):egaa095. 10.1093/petrology/egaa095

[CR29] Gerlach TM (2004) Volcanic sources of tropospheric ozone-depleting trace gases. Geochem Geophys Geosyst. 10.1029/2004GC000747

[CR30] Gerlach TM, Nordlie BE (1975) The COHS gaseous system; part II, temperature, atomic composition, and molecular equilibria in volcanic gases. Am J Sci 275:377–394

[CR31] Ghiorso MS, Gualda GAT (2015) An H_2_O-CO_2_ mixed fluid saturation model compatible with rhyolite-MELTS. Contrib Mineral Petrol 169:1–30. 10.1007/s00410-015-1141-8

[CR32] GorojovskyLR, Wood BJ (2026) Solubility and speciation of sulfur in silicate melts under crustal conditions. Earth Planet Sci Lett 687:120088 10.1016/j.epsl.2026.120088

[CR33] Hanfland M, Beister H, Syassen K (1989) Graphite under pressure: equation of state and 1st-order Raman modes. Phys Rev B 39:12598–12603. 10.1103/PhysRevB.39.12598 10.1103/physrevb.39.12598 9948126

[CR34] Hauri E (2002) SIMS analysis of volatiles in silicate glasses, 2: isotopes and abundances in Hawaiian melt inclusions. Chem Geol 183:115–141. 10.1016/S0009-2541(01)00374-6

[CR35] Heald EF, Naughton JJ, Barnes IL (1963) Chemistry of volcanic gases. 2. Use of equilibrium calculations in interpretation of volcanic gas samples. J Geophys Res. 10.1029/JZ068i002p00545

[CR36] Holloway JR (1977) Fugacity and activity of molecular species in supercritical fluids. In: Thermodynamics in Geology: Proceedings of the NATO Advanced Study Institute held in Oxford, England, September 17–27, 1976, pp 161–181. Springer Netherlands, Dordrecht. 10.1007/978-94-010-1252-2_9

[CR37] Iwasaki B, Katsura T (1967) The solubility of hydrogen chloride in volcanic rock melts at a total pressure of one atmosphere and at temperatures of 1200°C and 1290°C under anhydrous conditions. Bull Chem Soc Jpn 40:554–561. 10.1246/bcsj.40.554

[CR38] Jakobsson S, Oskarsson N (1994) The system CO in equilibrium with graphite at high pressure and temperature: an experimental study. Geochim Cosmochim Acta 58:9–17. 10.1016/0016-7037(94)90442-1

[CR39] Klobas JE, Weisenstein DK, Salawitch RJ, Wilmouth DM (2020) Reformulating the bromine alpha factor and equivalent effective stratospheric chlorine (EESC): evolution of ozone destruction rates of bromine and chlorine in future climate scenarios. Atmos Chem Phys 20:9459–9471. 10.5194/acp-20-9459-2020

[CR40] Kress VC, Carmichael ISE (1991) The compressibility of silicate liquids containing Fe2O3 and the effect of composition, temperature, oxygen fugacity and pressure on their redox states. Contrib Mineral Petrol 108:82–92. 10.1007/BF00307328

[CR41] Mangan M, Sisson T (2000) Delayed, disequilibrium degassing in rhyolite magma: decompression experiments and implications for explosive volcanism. Earth Planet Sci Lett 183:441–455. 10.1016/S0012-821X(00)00299-5

[CR42] Martin RS, Mather TA, Pyle DM (2006) High-temperature mixtures of magmatic and atmospheric gases. Geochem Geophys Geosyst. 10.1029/2005GC001186

[CR43] Martin RS, Wheeler JC, Ilyinskaya E, Braban CF, Oppenheimer C (2012) The uptake of halogen (HF, HCl, HBr and HI) and nitric (HNO_3_) acids into acidic sulphate particles in quiescent volcanic plumes. Chem Geol 296:19–25. 10.1016/j.chemgeo.2011.12.013

[CR44] Metcalfe A, Moune S, Komorowski JC, Kilgour G, Jessop DE, Moretti R, Legendre Y (2021) Magmatic processes at La Soufrière de Guadeloupe: insights from crystal studies and diffusion timescales for eruption onset. Front Earth Sci 9:617294. 10.3389/feart.2021.617294

[CR46] Moore G, Vennemann T, Carmichael ISE (1998) An empirical model for the solubility of H_2_O in magmas to 3 kilobars. Am Mineral 83:36–42. 10.2138/am-1998-1-203

[CR47] Moussallam Y, Towbin WH, Plank T, Bureau H, Khodja H, Guan Y et al (2024) ND70 series basaltic glass reference materials for volatile element (H_2_O, CO_2_, S, Cl, F) measurement and the C ionisation efficiency suppression effect of water in silicate glasses in SIMS. Geostand Geoanal Res 48:637–660. 10.1111/ggr.12572

[CR45] Métrich N, Zanon V, Créon L, Hildenbrand A, Moreira M, Marques FO (2014) Is the ‘Azores hotspot’ a wetspot? Insights from the geochemistry of fluid and melt inclusions in olivine of Pico basalts. J Petrol 55:377–393. 10.1093/petrology/egt071

[CR48] Newman S, Lowenstern JB (2002) VolatileCalc: a silicate melt-H_2_O-CO_2_ solution model written in Visual Basic for Excel. Comput Geosci 28:597–604. 10.1016/S0098-3004(01)00081-4

[CR49] O’Neill HSC, Mavrogenes JA (2002) The sulfide capacity and the sulfur content at sulfide saturation of silicate melts at 1400 C and 1 bar. J Petrol 43:1049–1087. 10.1093/petrology/43.6.1049

[CR50] O’Neill HSC, Mavrogenes JA (2022) The sulfate capacities of silicate melts. Geochim Cosmochim Acta 334:368–382. 10.1016/j.gca.2022.06.020

[CR51] Pyle DM, Mather TA (2009) Halogens in igneous processes and their fluxes to the atmosphere and oceans from volcanic activity: a review. Chem Geol 263:110–121. 10.1016/j.chemgeo.2008.11.013

[CR52] Reid RC, Prausnitz JM, Poling BE (1987) The properties of gases and liquids, 4th edn. McGraw-Hill, New York

[CR53] Rusiecka MK, Wood BJ (2025) Chlorine and NaCl in hydrous basaltic melts. Geochim Cosmochim Acta 393:208–218. 10.1016/j.gca.2025.01.020

[CR54] Sandland TO, Du LS, Stebbins JF, Webster JD (2004) Structure of Cl-containing silicate and aluminosilicate glasses: a 35Cl MAS-NMR study. Geochim Cosmochim Acta 68:5059–5069. 10.1016/j.gca.2004.07.017

[CR55] Scholtysik R, Canil D (2021) The effects of S, Cl and oxygen fugacity on the sublimation of volatile trace metals degassed from silicate melts with implications for volcanic emissions. Geochim Cosmochim Acta 301:141–157. 10.1016/j.gca.2021.02.018

[CR56] Sharpe MS, Barker SJ, Rooyakkers SM, Wilson CJ, Chambefort I, Rowe MC et al (2022) A sulfur and halogen budget for the large magmatic system beneath Taupō volcano. Contrib Mineral Petrol 177:95. 10.1007/s00410-022-01959-w

[CR57] Shinohara H (2009) A missing link between volcanic degassing and experimental studies on chloride partitioning. Chem Geol 263:51–59. 10.1016/j.chemgeo.2008.12.001

[CR58] Shishkina TA, Botcharnikov RE, Holtz F, Almeev RR, Portnyagin MV (2010) Solubility of H_2_O- and CO_2_-bearing fluids in tholeiitic basalts at pressures up to 500 MPa. Chem Geol 277:115–125. 10.1016/j.chemgeo.2010.07.014

[CR79] Signorelli S, Carroll M (2002) Experimental study of Cl solubility in hydrous alkaline melts: constraints on the theoretical maximum amount of Cl in trachytic and phonolitic melts. Contrib Mineral Petrol 143:209–218 10.1007/s00410-001-0320-y

[CR59] Signorelli S, Carroll MR (2000) Solubility and fluid-melt partitioning of Cl in hydrous phonolitic melts. Geochim Cosmochim Acta 64:2851–2862. 10.1016/S0016-7037(00)00386-0

[CR60] Spilliaert N, Allard P, Métrich N, Sobolev AV (2006) Melt inclusion record of the conditions of ascent, degassing, and extrusion of volatile-rich alkali basalt during the powerful 2002 flank eruption of Mount Etna (Italy). J Geophys Res Solid Earth. 10.1029/2005JB003934

[CR61] Stanley BD, Hirschmann MM, Withers AC (2011) CO_2_ solubility in Martian basalts and Martian atmospheric evolution. Geochim Cosmochim Acta 75:5987–6003. 10.1016/j.gca.2011.07.027

[CR62] Stebbins JF, Du LS (2002) Chloride ion sites in silicate and aluminosilicate glasses: a preliminary study by ^35^Cl solid-state NMR. Am Mineral 87:359–363. 10.2138/am-2002-2-320

[CR63] Stelling J, Botcharnikov RE, Beermann O, Nowak M (2008) Solubility of H_2_O- and chlorine-bearing fluids in basaltic melt of Mount Etna at T = 1050–1250°C and P = 200 MPa. Chem Geol 256:102–110. 10.1016/j.chemgeo.2008.04.009

[CR64] Symonds RB, Rose WI, Bluth GJ, Gerlach TM (1994) Volcanic-gas studies: methods, results, and applications. Rev Mineral 30:1–1

[CR67] Thomas RW, Wade J, Wood BJ (2023) The bonding environment of chlorine in silicate melts. Chem Geol 617:121269. 10.1016/j.chemgeo.2022.121269

[CR65] Thomas RW, Wood BJ (2021) The chemical behaviour of chlorine in silicate melts. Geochim Cosmochim Acta 294:28–42. 10.1016/j.gca.2020.11.018

[CR66] Thomas RW, Wood BJ (2023) The effect of composition on chlorine solubility and behavior in silicate melts. Am Mineral 108:814–825. 10.2138/am-2022-8450

[CR68] von Glasow R, Bobrowski N, Kern C (2009) The effects of volcanic eruptions on atmospheric chemistry. Chem Geol 263:131–142. 10.1016/j.chemgeo.2008.08.020

[CR69] Webster JD (1992) Water solubility and chlorine partitioning in Cl-rich granitic systems: effects of melt composition at 2 kbar and 800°C. Geochim Cosmochim Acta 56:679–687. 10.1016/0016-7037(92)90089-2

[CR73] Webster JD, Baker DR, Aiuppa A (2018) Halogens in mafic and intermediate-silica content magmas. In: Harlov D, Aranovich L (eds) The Role of Halogens in Terrestrial and Extraterrestrial Geochemical Processes. Springer Geochemistry. Springer. 10.1007/978-3-319-61667-4_6

[CR70] Webster JD, De Vivo B (2002) Experimental and modeled solubilities of chlorine in aluminosilicate melts, consequences of magma evolution, and implications for exsolution of hydrous chloride melt at Mt. Somma-Vesuvius. Am Mineral 87:1046–1061. 10.2138/am-2002-8-902

[CR71] Webster JD, Kinzler RJ, Mathez EA (1999) Chloride and water solubility in basalt and andesite melts and implications for magmatic degassing. Geochim Cosmochim Acta 63:729–738. 10.1016/S0016-7037(99)00043-5

[CR72] Webster JD, Vetere F, Botcharnikov RE, Goldoff B, McBirney A, Doherty AL (2015) Experimental and modeled chlorine solubilities in aluminosilicate melts at 1 to 7000 bars and 700 to 1250°C: applications to magmas of Augustine Volcano, Alaska. Am Mineral 100:522–535. 10.2138/am-2015-5014

[CR74] Witham F, Blundy J, Kohn SC, Lesne P, Dixon J, Churakov SV, Botcharnikov R (2012) SolEx: a model for mixed COHSCl-volatile solubilities and exsolved gas compositions in basalt. Comput Geosci 45:87–97. 10.1016/j.cageo.2011.09.021

[CR75] Witter JB, Kress VC, Newhall CG (2005) Volcán Popocatépetl, Mexico. Petrology, magma mixing, and immediate sources of volatiles for the 1994-present eruption. J Petrol 46:2337–2366. 10.1093/petrology/egi058

[CR76] Zhao L, Tang J, Zhou M, Shen K (2022) A review of the coefficient of thermal expansion and thermal conductivity of graphite. New Carbon Mater 37:544–555. 10.1016/S1872-5805(22)60603-6

[CR77] Zurek J, Moune S, Williams-Jones G, Vigouroux N, Gauthier PJ (2019) Melt inclusion evidence for long term steady-state volcanism at Las Sierras-Masaya volcano, Nicaragua. J Volcanol Geotherm Res 378:16–28. 10.1016/j.jvolgeores.2019.04.007

