# Supplementary material for: Chlorine (Cl) and hydrogen chloride (HCl) solubility in hydrous silicate melts: implications for volcanic gas composition
Source: Contrib Mineral Petrol. 2026 May 22;181(6):46. doi: 10.1007/s00410-026-02332-x (PMC13194342; doi:10.1007/s00410-026-02332-x)
Supplement: Supplementary file 1 — Supplementary Material 1 [file 410_2026_2332_MOESM1_ESM.pdf]

Supplementary Online Material for "Chlorine (Cl) and hydrogen chloride (HCl) solubility in hydrous silicate melts: implications for volcanic gas composition" by Rusiecka, M.K., and Wood, B.J., Contributions to Mineralogy and Petrology

Equations used for the MRK gas speciation:

Chlorine

Chloride capacity (this study):

$$logC_{Cl} = 1.15 + \frac{4359X_{Ca} - 3055X_{Si} + 2059X_{Fe} - 3875X_K + 163X_{Mg} - 514P}{T}$$

T in Kelvin, P in GPa, X_M on single cation basis.

HCl fugacity (this study):

$$f_{\text{HCl}} = K_{\text{HCl}} \times \frac{(f_{\text{H}_2\text{O}})^{0.5}}{(K_{\text{H}_2\text{O}})^{0.5}} \times \frac{\text{Cl (wt.\%)}}{C_{\text{Cl}}}$$

$$\log_{10} K_{H_2O} = \frac{12850}{T} - 2.8675$$

$$\log_{10} HCl = \frac{4894.6}{T} + 0.3436$$

Water

Moore (1998):

a = 2565; b_Al_2O3 = -1.997; b_FeO_{tot} = -0.9275; b_Na_2O = 2.736; c = 1.171; d = -14.21;

$$\ln f H_2O = \frac{2 \log XH_2O - \frac{a}{T} - \sum_i bX_i \frac{P}{T} - d}{c}$$

P in bars, T in Kelvin, X_i on anhydrous basis.

Burnham (1994):

$$(a_w^m)_{P,T,X_w^m \leq 0.5} = k_w (X_w^m)^2$$

$$(a_w^m)_{P,T,X_w^m > 0.5} = 0.25 k_w \exp\left(\frac{6.52 - \frac{2667}{T}}{X_w^m - 0.5}\right)$$

$$lnk_w = 5.00 + lnP(4.481 \times 10^{-8}T^2 - 1.51 \times 10^{-4}T - 1.137) + (lnP)^2(1.831 \times 10^{-8}T^2 - 4.882 \times 10^{-5}T + 0.04656) + 7.8 \times 10^{-3}(lnP)^3 - 5.012 \times 10^{-4}(lnP)^4 + T(4.754 \times 10^{-3} - 1.621 \times 10^{-6}T)$$

P in bars, t in Kelvin, X on eight oxygen basis.

Sulphur

Sulphide and sulphate capacities (Gorojovsky and Wood 2025)

$\log C_{S^{2-}} = 0.65 + \big(-3368X_{Si_{0.5}O} - 1233X_{Al_{0.6}O} + 1295X_{CaO} + 44885X_{K_2O} + 10914X_{FeO} \cdot X_{Si_{0.5}O} - 871864X_{FeO} \cdot X_{K_2O} - 225569X_{FeO} \cdot X_{Na_2O} + 54392X_{FeO} \cdot X_{Al_{0.5}O} - 7585\big) / \mathrm{T} + 3.9\,\mathrm{erf}[X_{FeO}]$

$logC_{S^{6+}} = 196.0 + \big(-7633X_{Si_{0.5}O} - 10670_{Ti_{0.5}O} - 6901X_{Al_{0.6}O} - 4625X_{FeO_T} + 19114X_{MnO} + 11740X_{CaO} + 33267X_{Na_2O} - 4095\big) /\ T\ - 57.3\log{(T)}$

Reaction constants (Bouillung and Wood 2021, 2023)

$\log_{10} K_{SO_2} = \frac{18880}{T} - 3.8018$

$\log_{10} K_{H_2S} = \frac{27103}{T} - 4.1973$

Effect of P (Thomas and Wood 2026)

$$\frac{\delta logC_{S^{2-}}}{\delta P} = \frac{-0.056(\pm 0.008)}{T}$$

$$\frac{\delta logC_{S^{6+}}}{\delta P} = \frac{-0.165(\pm 004)}{T}$$

T in Kelvin, P in bars, X_M on single oxygen basis.

Oxygen

FMQ base (Frost 1991)

$logfO_2 = \frac{-25096.3}{T} + 8.735 + 0.110\frac{(P-1)}{T}$

NNO base (Frost 1991)

$logfO_2 = \frac{-24930}{T} + 9.36 + 0.046\frac{(P-1)}{T}$

CCO base (Jakobsson and Oskarsson 1994)

$logfO_2 = \frac{-21803}{T} + 4.325 + 0.171\frac{(P-1)}{T}$

P in bars, T in Kelvin.

Carbon

CH4 equilibrium (Ohmoto and Kerrick 1977)

$CH_4 + 2O_2 = CO_2 + 2H_2O$

$\log_{10} K_{CH_4} = \frac{41997}{T} + 0.719\log_{10}T - 2.404$

$Ptrans\ (kbar) = 0.025T + 12.592$ (Day 2012)

if Pkbar<Ptrans

$\log_{10} K1 = 40.07639 - 2.53932 \times 10^{-2}T + 5.27096 \times 10^{-6}T^2 + 0.0267\frac{(P(kbar)-1)}{T}$

(Holloway et al. 1992)

If Pkbar >= Ptrans:

$\log_{10} K1 = 0.2450 - \frac{2.027\times10^4}{T} - \frac{4.730\times10^4}{T^2} + 0.0267\frac{(1.949\times10^{-7}P(Pa)-10^6)}{T}$ (Duncan and Dasgupta 2017)

$$lnXCO_3^{2-} = -\frac{2.384\times10^{-5}P(Pa)}{RT} + \frac{-1.6448\times10^5}{RT} + \frac{1.4732\times10^3 lnfCO_2}{T} + \frac{-43.6385}{R} + 3.291NBO + \frac{(1.68\times10^5 XCaO + 1.7590\times10^5 XNa_2O + 2.1085\times10^5 XK_2O)}{RT}$$

Dissolved carbon (Eguchi and Dasgupta 2018)

$$lnXCO_2 = -\frac{1.9244\times10^{-5}P(Pa)}{RT} + \frac{-9.0212\times10^4}{RT} + \frac{1.1149\times10^3 fCO_2}{T} + \frac{-43.0815}{R} - 7.0937NBO$$

$$wt.\%\ CO_3^{2-} = [\frac{44.01\times XCO_3^{2-}}{44.01\times XCO_3^{2-} + (1-(XCO_3^{2-}+XCO_2)FWone}] \times 100$$

$$wt.\%\ CO_2 = [\frac{44.01\times XCO_2}{44.01\times XCO_2 + (1-(XCO_3^{2-}+XCO_2)FWone}] \times 100$$

FWone = one-oxygen formula weight of one mol of volatile free melt in g/mol

NBO = non bridging oxygens

If NBO > 0.5, wt. % CO_2 = 0

$$wt.\%\ CO_{2_{tot}} = wt.\%CO_3^{2-} + wt.\%CO_2$$

Day, H. W. (2012). A revised diamond-graphite transition curve. American Mineralogist, 97(1), 52-62. https://doi.org/10.2138/am.2011.3763

Duncan, M. S. and R. Dasgupta. (2017) Rise of Earth's atmospheric oxygen controlled by efficient subduction of organic carbon. Nat. Geosci., 10, 387. https://doi.org/10.1038/ngeo2939

Eguchi, J., & Dasgupta, R. (2018). A CO_2 solubility model for silicate melts from fluid saturation to graphite or diamond saturation. Chemical Geology, 487, 23-38. https://doi.org/10.1016/j.chemgeo.2018.04.012

Frost, B. R. (1991). Introduction to oxygen fugacity and its petrologic importance. In Oxide minerals (pp. 1-10). De Gruyter. https://doi.org/10.1515/9781501508684-004

Gorojovsky, L. R., & Wood, B. J. (2025). Solubility and speciation of sulfur in silicate melts under crustal conditions. EarthArxiv https://doi.org/10.31223/X5T755

Holloway, J. R., Pan, V., & Gudmundsson, G. (1992). High-pressure fluid-absent melting experiments in the presence of graphite: oxygen fugacity, ferric/ferrous ratio and dissolved CO2. European Journal of Mineralogy, 4(1), 105-114. 10.1127/ejm/4/1/0105

Jakobsson, S., & Oskarsson, N. (1994). The system CO in equilibrium with graphite at high pressure and temperature: An experimental study. Geochimica et Cosmochimica Acta, 58(1), 9-17. https://doi.org/10.1016/0016-7037(94)90442-1

Kress, V. C., & Carmichael, I. S. (1991). The compressibility of silicate liquids containing Fe_2O_3 and the effect of composition, temperature, oxygen fugacity and pressure on their redox states. Contributions to Mineralogy and Petrology, 108(1), 82-92. https://doi.org/10.1007/BF00307328

Moore, G., Vennemann, T., & Carmichael, I. S. E. (1998). An empirical model for the solubility of H_2O in magmas to 3 kilobars. American Mineralogist, 83(1-2), 36-42. https://doi.org/10.2138/am-1998-1-203

Ohmoto, H., & Kerrick, D. M. (1977). Devolatilization equilibria in graphitic systems. American Journal of Science, 277(8), 1013–1044. https://doi.org/10.2475/ajs.277.8.1013

Thomas, R.W, Wood, B.J. Sulfur speciation in silicate melts at high pressure. Under Review in GCA

Attainment of equilibrium

Figure illustrating homogenous distribution of Cl in the experimental glass sample. The profile (red line) on the right panel corresponds with composition profile in the left panel (red line on the SE image). Sample DCB-2, Brothers volcano dacite, 1200 °C, 1.63 GPa, 2 hours.

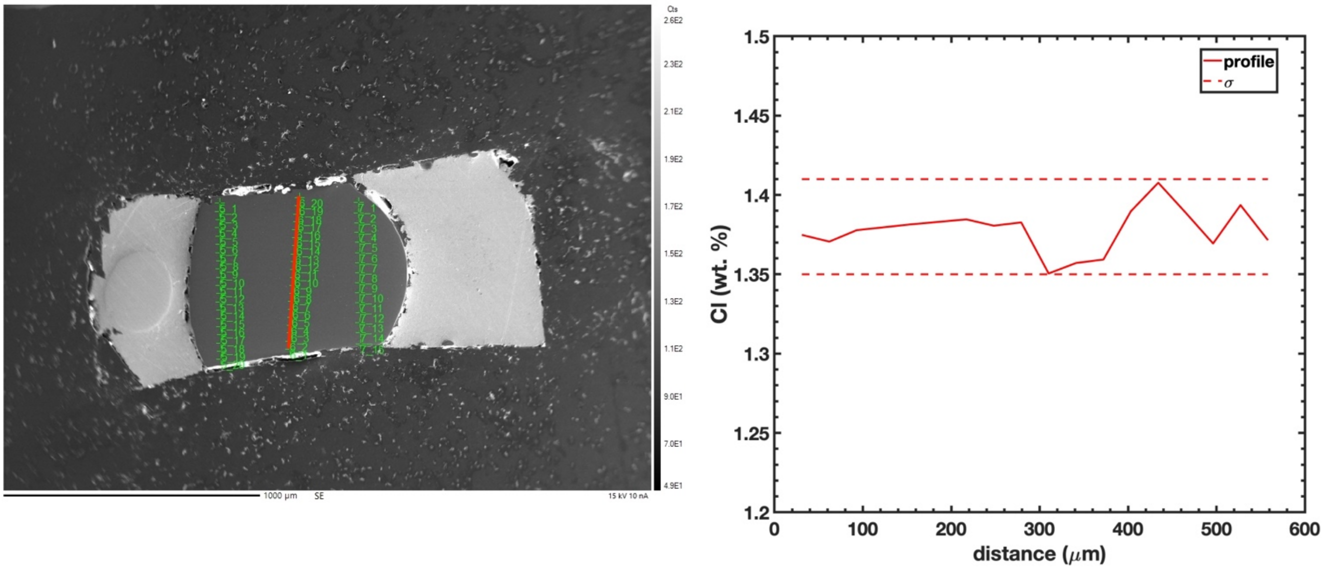

Table showing representative analyses of the AgI/Cl buffer

Na	*Mg*	*Si*	*Al*	*K*	*Ca*	*Fe*	*Cl*	*Ag*	*I*	*Pt*	*Total*
-0.0106	-0.565	0.0439	0.108	-0.071	-0.05	-0.005	4.411	62.17	37.03	-0.086	102.97
0.032	-0.527	0.007	0.124	-0.057	-0.07	-0.048	4.37	58.92	38.89	0.0856	101.73
0.1325	-0.503	0.0325	0.145	-0.076	-0.01	-0.008	3.613	56.6	41.27	0.1243	100.48
0.2356	-0.437	0.0223	0.08	-0.088	-0.05	0.063	3.479	57.89	41.89	0.1105	99.235
0.2405	-0.534	0.0367	0.091	-0.02	0	-0.01	3.536	57.26	41.79	0.2627	97.989
